# Regulation of *PHOX2B* gene expression by the long non‐coding natural antisense RNA *PHOX2B‐AS1*

**DOI:** 10.1111/febs.70410

**Published:** 2026-01-24

**Authors:** Simona Di Lascio, Ana Lucia Cuadros Gamboa, Martina Bertocchi, Filippo Chiesa, Francesca Cargnin, Ettore Mosca, Paride Pelucchi, Viviana Tritto, Stefania Corti, Isabella Ceccherini, Paola Riva, Roberta Benfante, Diego Fornasari

**Affiliations:** ^1^ Department of Medical Biotechnology and Translational Medicine Università degli Studi di Milano Milan Italy; ^2^ Department of Pediatrics, Papé Family Pediatric Research Institute Oregon Health & Science University Portland OR USA; ^3^ Institute of Biomedical Technologies National Research Council Segrate (Milan) Italy; ^4^ Department of Pathophysiology and Transplantation, Dino Ferrari Center University of Milan Milan Italy; ^5^ Neurology Unit Foundation IRCCS Ca' Granda Ospedale Maggiore Policlinico Milan Italy; ^6^ Center for Preclinical Research Foundation IRCCS Ca' Granda Ospedale Maggiore Policlinico Milan Italy; ^7^ UOSD Aggregation Area of Research Laboratories IRCCS Istituto Giannina Gaslini Genoa Italy; ^8^ CNR – Neuroscience Institute Vedano al Lambro (MB) Italy; ^9^ NeuroMi – Milan Center for Neuroscience University of Milano Bicocca Milan Italy; ^10^ Present address: Human Technopole Viale Rita Levi‐Montalcini 1 Milan 20157 Italy

**Keywords:** congenital central hypoventilation syndrome, lncRNA, natural antisense transcript, neuroblastoma, PHOX2B

## Abstract

Paired mesoderm homeobox protein 2B (PHOX2B) is a transcription factor essential for autonomic nervous system development. Heterozygous mutations in the *PHOX2B* gene are associated with neurodevelopmental disorders, including congenital central hypoventilation syndrome and Hirschsprung's disease. Additionally, *PHOX2B* plays a role in the genetic landscape of neuroblastoma, with mutations detected in both familial and sporadic forms of this rare cancer. Notably, *PHOX2B* is highly expressed in most neuroblastoma cells. Despite its significance, little is known about the regulation of *PHOX2B* gene expression, and limited attention has been given to the genomic features of the antisense strand at the *PHOX2B* locus, although the presence of an antisense transcript is suggested by bioinformatics analyses. In this study, we characterize the recently annotated human antisense transcript *PHOX2B‐AS1* and the previously unidentified mouse antisense *Phox2b* transcript. Our findings reveal that PHOX2B positively regulates *PHOX2B‐AS1* expression and that inhibiting the antisense transcript reduces PHOX2B protein levels. Together, these results provide strong evidence for the existence of a gene antisense to *PHOX2B* and highlight a strict correlation and reciprocal regulation between *PHOX2B* and *PHOX2B‐AS1*.

## Graphical abstract

PHOX2B is a transcription factor essential for autonomic nervous system development. We identify and characterize *PHOX2B‐AS1*, a human long non‐coding antisense transcript at the *PHOX2B* locus, along with its murine counterpart. Our findings reveal bidirectional transcription and reciprocal regulation: PHOX2B activates *PHOX2B‐AS1*, whereas *PHOX2B‐AS1* inhibition reduces PHOX2B protein levels. This highlights a critical regulatory interplay between sense and antisense transcription at the *PHOX2B* locus.

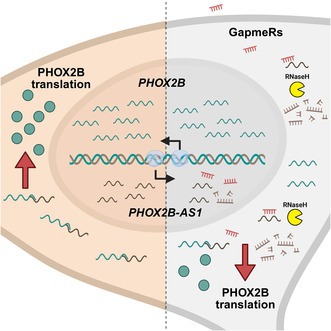

**Abbreviations**
3‐KDG: 3‐keto‐desogestrel
ANS: autonomic nervous system
ASO: antisense oligonucleotide
ATRA: all‐trans retinoic acid
CAGE: cap analysis of gene expression
CCHS: congenital central hypoventilation syndrome
ChIP‐Seq: chromatin immunoprecipitation sequencing
DMEM: Dulbecco's modified Eagle's medium
epiVII: epifacial neurons
GFR: growth factor reduced
HD: homeodomain
HiPSCs: human‐induced pluripotent stem cells
HSCR: Hirschsprung's disease
IGV: Integrated Genome Viewer
invSINEB2: inverted short interspersed nuclear element B2
ISH: *in situ* hybridization
Lc: locus coeruleus
lncRNA: long non‐coding RNA
nA: nucleus ambiguus
NAT: natural antisense transcript
NB: neuroblastoma
NCC: neural crest cell
ncRNA: non‐protein‐coding RNA
NTS: nucleus of solitary tract
nV: trigeminal motor nucleus
ORF: open reading frame
PARM: polyAla repeat expansion mutation
PFA: paraformaldehyde
qPCR: quantitative PCR
RACE: rapid amplification of cDNA ends
SAP: sympathoadrenal progenitor
SD: standard deviation
Sp5: spinal trigeminal tract
SS‐RT‐PCR: strand‐specific RT‐PCR
SymNs: sympathetic neurons
TPM: transcripts per million
TSS: transcription start site
Ves: vestibular neurons
VII: facial motor nucleus

## Introduction

Emerging evidence suggests that the human genome contains more genes encoding regulatory non‐protein‐coding RNAs (ncRNAs) than those encoding proteins [1, 2]. One major class of regulatory RNA genes includes the class of RNAs longer than 200 nucleotides, which are generally classified as long non‐coding RNAs (lncRNAs), of which natural antisense transcripts (NATs) represent a subgroup. NATs are RNA molecules transcribed from the DNA strand opposite to that of sense transcripts. They partially overlap with sense genes or their regulatory regions and can regulate gene expression positively or negatively at various levels by means of multiple mechanisms [3, 4, 5, 6]. The existence of a human NAT that is transcribed opposite the gene encoding *PHOX2B* has been recently annotated in Ensembl (https://www.ensembl.org) and GenBank (https://www.ncbi.nlm.nih.gov) databases, and in this study, we provide a detailed functional characterization of this antisense transcript.

The *PHOX2B* gene encodes a crucial highly conserved homeodomain transcription factor essential for the development of neural crest cells into the autonomic nervous system (ANS) and several brainstem nuclei responsible for autonomic control of breathing [7, 8, 9, 10]. Complete inactivation of the *Phox2b* gene and consequent loss of PHOX2B protein expression are embryonically lethal in all rodent models in which it has been studied and no *PHOX2B*‐null human has been described [7, 9]. Mutations in the *PHOX2B* gene and/or dysregulation of its expression are associated with several isolated or syndromic disorders of ANS development. Heterozygous dominant mutations in *PHOX2B* cause Congenital Central Hypoventilation Syndrome (CCHS, MIM# 209880) [11, 12] with causative variants including missense, nonsense, frameshift, and polyAla repeat expansion mutations (PARMs). The latter involve in‐frame triplet duplications within the 20‐residue polyalanine stretch at the C‐terminus, leading to the incorporation of 4–13 additional alanines, and are the most frequent mutations among isolated CCHS patients. This rare neurodevelopmental disease is characterized by a compromised ventilatory response to hypoxia and hypercapnia, necessitating artificial ventilation for life support. Additionally, due to the critical role of *PHOX2B* in early ANS development, CCHS embodies a wide range of symptoms associated with ANS dysfunction [13]. CCHS can occur as an isolated condition or in conjunction with other neural crest‐derived disorders including neuroblastoma (NB), a tumor of the sympathetic nervous system, and Hirschsprung's disease (HSCR), or aganglionic megacolon. *PHOX2B* causative mutations have been documented in NB and HSCR, alongside the recognition that common variants of *PHOX2B* contribute as predisposing genetic factors [14, 15]. *PHOX2B* mutations are also a rare cause of non‐syndromic NB [16, 17, 18, 19]. Moreover, *PHOX2B* overexpression has been observed in both NB cell lines and in tumor samples derived from NB patients [20] with elevated *PHOX2B* levels correlating with a poorer prognosis [21, 22]. These observations are reinforced by recent findings identifying *PHOX2B* as a key member of the core regulatory circuitry of transcription factors that defines adrenergic NB identity and promotes tumorigenesis [23, 24].

Despite its critical role, the regulatory mechanisms governing the transcriptional and post‐transcriptional control of *PHOX2B* remain largely unexplored. At the transcriptional level, PHOX2A acts as a transcriptional regulator of *PHOX2B* [25] which in turn regulates itself by a positive regulatory loop [26]. At the post‐transcriptional level, *PHOX2B* expression is negatively regulated by miR‐204 [27], miR‐99b‐5p [28] and miR‐125a [29]. In NB patients, reduced levels of these miRNAs, by contributing to *PHOX2B* overexpression, correlate with poor patient prognosis and have a role in chemotherapy resistance. This suggests that *PHOX2B*, through miRNA‐mediated regulation, plays a role in apoptosis and differentiation in NB, highlighting the potential for targeting this transcription factor at the post‐transcriptional level.

In this study, we reveal that the *PHOX2B* locus undergoes bidirectional transcription in both human cell lines and mouse brain tissues. Our findings show that the tissue expression pattern of *PHOX2B* NAT closely mirrors that of the sense *PHOX2B* gene, suggesting a potentially significant regulatory role. Importantly, we provide evidence that the NAT regulates *PHOX2B* expression at the post‐transcriptional level, uncovering an additional layer of *PHOX2B* gene expression control.

## Results

### Genomic organization and bidirectional transcription at the human *PHOX2B* gene locus

**Fig. 1:**
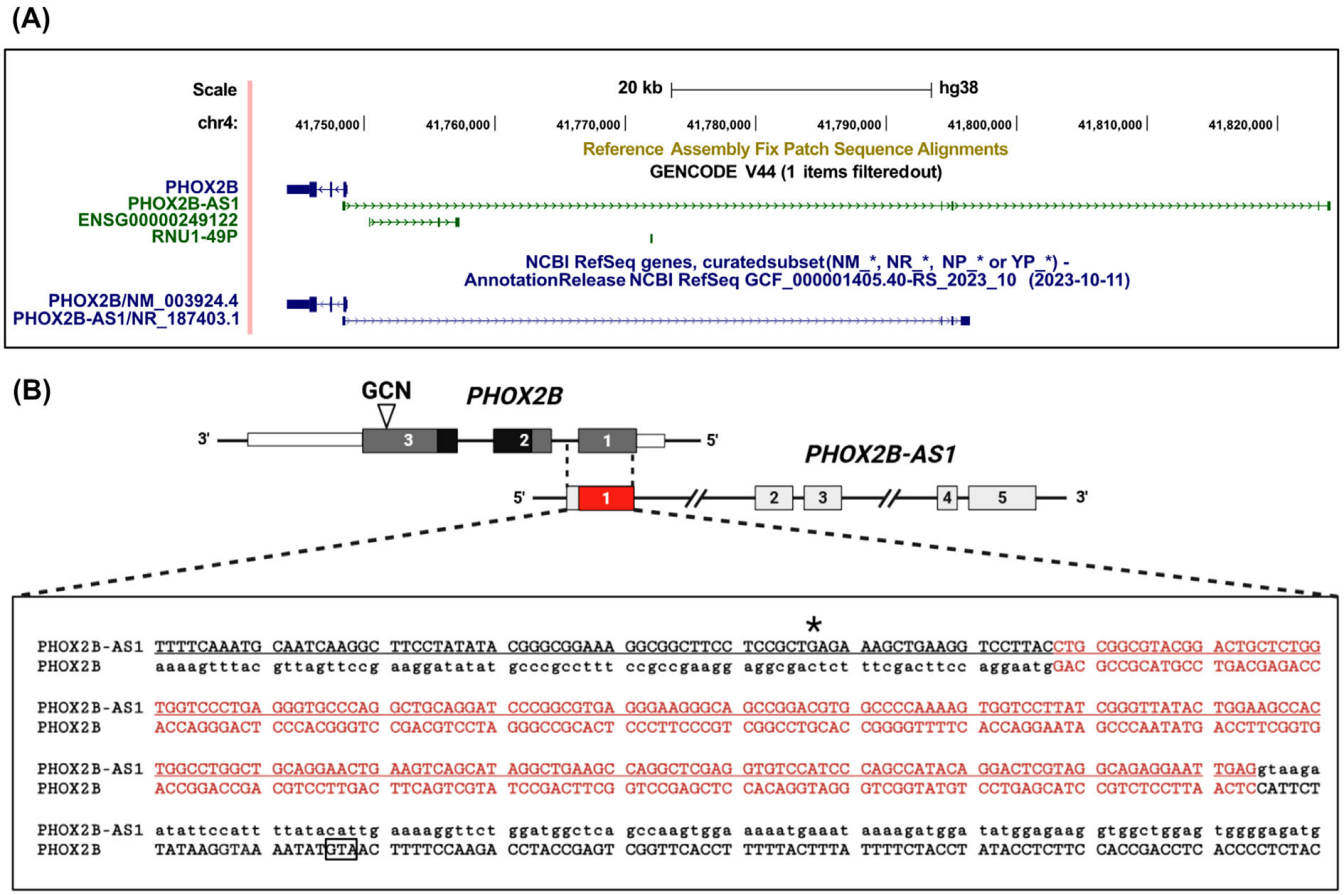
Alignment of *PHOX2B* with complementary *PHOX2B‐AS1* sequence. (A) Genomic location of the *PHOX2B* and *PHOX2B‐AS1* transcripts as annotated in the UCSC Genome Browser (https://genome.ucsc.edu; GRCh38.p14 assembly). GENCODE v44 (https://www.gencodegenes.org/) annotation identifies the *PHOX2B* (ENST00000226382.4) and *PHOX2B‐AS1* (ENST00000508038.1) transcripts according to Ensembl release 110 while NCBI annotation reports *PHOX2B* (NM_003924.4) and *PHOX2B‐AS1* (NR_187403.1) transcripts. (B) Schematic representation of *PHOX2B* and *PHOX2B‐AS1*. The sequence shown corresponds to the region encompassing the first exons of both transcripts, with the two strands manually annotated according to Ensembl. The underlined sequence represents the first exon of *PHOX2B‐AS1* (ENST00000508038.1), whereas the asterisk marks the first nucleotide of *PHOX2B‐AS1* as annotated in the GenBank database (NR_187403.1). Uppercase letters indicate exon regions; lowercase letters indicate intronic sequences. The putative overlapping region between mature transcripts is highlighted in red, and the PHOX2B translation start site (ATG) is boxed.

The *PHOX2B* gene (GenBank reference number NG_008243.1, Gene ID: 8929) is located on human chromosome 4 (4p14) and consists of three exons (Fig. 1A). It encodes a transcription factor of 314 amino acids, with a conserved 60‐residue DNA binding homeodomain (HD) and a stretch of 20 alanine residues at the C‐terminus. The HD is encoded by a DNA region spanning exons 2 and 3 (Fig. 1B, black boxes) and the GCN tract encoding for the polyalanine stretch is located in exon 3 (Fig. 1B, white triangle). An antisense gene transcribed in the opposite direction of *PHOX2B* was first annotated as *PHOX2B‐AS1* in the Ensembl database (ENSG00000250467) and more recently in the GenBank database (ref number NC_000004.12, gene ID 105374425) (Fig. 1A). This antisense transcript has also been identified by the FANTOM5 consortium (http://fantom.gsc.riken.jp/5), which has mapped transcription start sites across nearly a thousand human primary cells, cell lines, and tissues to provide a comprehensive overview of mammalian gene expression [30]. Notably, the expression profile of *PHOX2B‐AS1* closely correlates with that of *PHOX2B*, suggesting a potential regulatory relationship. According to Ensembl annotations (releases 100–112), *PHOX2B‐AS1* is a 907‐nucleotide transcript composed of five exons (ENST00000508038.1). In contrast, the GenBank database predicts five transcript variants of varying lengths (XR_001741668.2; XR_925256.3; XR_001741669.2; XR_001741670.2; XR_001741671.2) and has validated a transcript of 1240 nucleotides consisting of four exons (NR_187403.1). Notably, neither the predicted nor validated transcripts fully match the annotation in Ensembl. However, a portion of the first exon, as well as the second and third exons, are 100% conserved across these annotations (Fig. 1A and Fig. S1).

Both Ensembl and GenBank annotations indicate that *PHOX2B‐AS1* transcript initiates within the first intron of the *PHOX2B* gene. The antisense first exon contains 77 nucleotides (according to Ensembl) or 21 nucleotides (according to NCBI) that are complementary to the first intron of the *PHOX2B* gene. Additionally, it overlaps with the last 217 nucleotides of the first exon of *PHOX2B* mRNA, excluding the AUG start codon (Fig. 1B).

The latest Ensembl release (113, October 2024) has recently annotated 21 transcript variants, which we have included for completeness in Figs S2 and S3. However, our analysis is based on previous Ensembl releases, as all data presented here were generated before the most recent annotation. Notably, transcript variants ENST00000508038.2 (*PHOX2B‐AS1‐201*) and ENST00000819364.1 (*PHOX2B‐AS1‐214*) share 93.2% and 100% similarity, respectively, with previously annotated transcripts in the Ensembl and NCBI databases. Transcript variants ENST00000510602.1 (*PHOX2B‐AS1‐202*), ENST00000819366.1 (*PHOX2B‐AS1‐216*), ENST00000819367.1 (*PHOX2B‐AS1‐217*), ENST00000819368.1 (*PHOX2B‐AS1‐218*), ENST00000819369.1 (*PHOX2B‐AS1‐219*), and ENST00000819370.1 (*PHOX2B‐AS1‐220*) exhibit high similarity to the previously annotated gene ENSG00000249122 shown in Fig. 1A. The remaining transcript variants display varying degrees of similarity with previously annotated transcripts in the Ensembl and NCBI databases, with the highest level of conservation observed in the first three exons. Translation and Kozak sequence analysis of the *PHOX2B‐AS1* transcript predicts that it is non‐protein coding, as the probability of any start codon within its sequence functioning as an initiation codon is very low (Fig. S4).

**Fig. 2:**
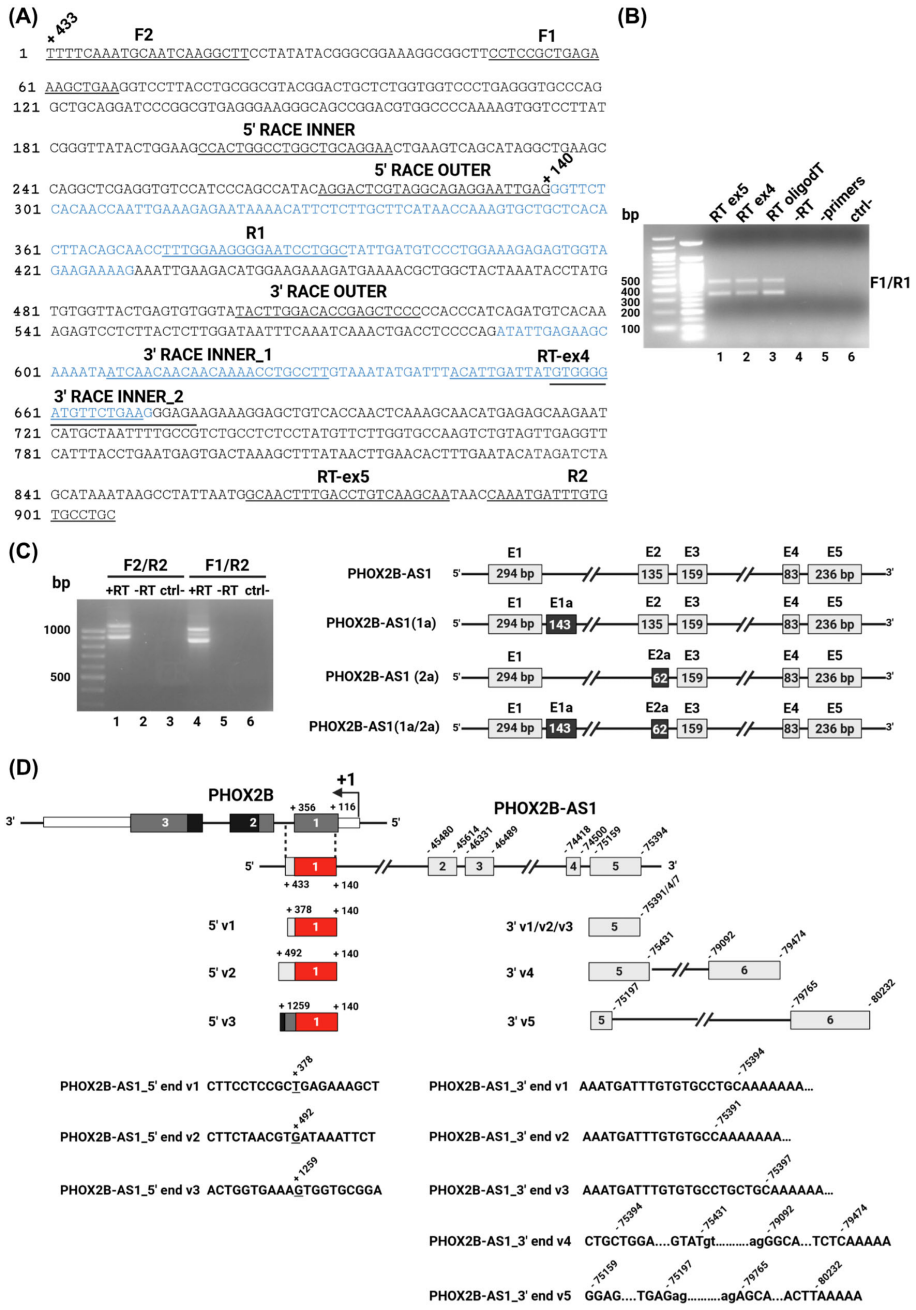
Identification and characterization of *PHOX2B‐AS1*. (A) Nucleotide sequence of the *PHOX2B‐AS1* transcript as annotated in the Ensembl database (ENST00000508038.1). Exons are indicated by alternating black and blue nucleotide sequences. Underlined sequences represent primers used in SS‐RT‐PCR (Strand‐Specific Reverse Transcription Polymerase Chain Reaction) analysis (panel B), conventional PCR analyses (panel C), and 5′ and 3′ RACE (Rapid Amplification of cDNA Ends) experiments (panel D). (B) SS‐RT‐PCR analysis of the *PHOX2B‐AS1* transcript. RNA from IMR32 was reverse transcribed using strand‐specific primers AS1 RT‐ex4 and AS1 RT‐ex5, or an oligodT (as positive control). Negative controls included RNA reverse transcribed without primers or without the RT enzyme. PCR products amplified using F1/R1 primers were analyzed on a 1% agarose gel. (C) PCR analysis and identification of *PHOX2B‐AS1* splice variants by sequencing, with schematic representations of the identified variants shown on the right. (D) 5′ and 3′ RACE analysis summarizing the identified alternative 5′ (underlined) and 3′ transcript ends.

To investigate the expression of an antisense transcript at the *PHOX2B* locus, we performed strand‐specific RT‐PCR (SS‐RT‐PCR) using RNA from the neuroblastoma cell line IMR32 and the primers indicated in Fig. 2A. This analysis yielded two bands of approximately 300 and 500 nucleotides, confirming the presence of multiple antisense transcripts (Fig. 2B). We subsequently amplified the predicted full‐length *PHOX2B‐AS1* from IMR32 cells, cloned the obtained bands, and verified the transcript identities by sequencing. Sequence analysis revealed four splicing variants, distinguished by the presence of an additional exon between exon 1 and exon 2 (named exon 1a) or a shorter version of exon 2 (named exon 2a) (Fig. 2C and Fig. S5). The newly identified exons were flanked by canonical AG‐GT splice acceptor and donor sites, and the final exon contained an AAUAAA polyadenylation signal. Notably, exon 1a is present in the following transcripts recently annotated in Ensembl: ENST00000819356.1 (*PHOX2B‐AS1‐206*), ENST00000819357.1 (*PHOX2B‐AS1‐207*), ENST00000819360.1 (*PHOX2B‐AS1‐210*), and ENST00000819362.1 (*PHOX2B‐AS1‐212*).

5′‐ and 3′‐rapid amplification of cDNA ends (RACE) analyses confirmed that the identified boundaries correspond to the predicted transcript while also revealing the presence of alternative 5′ and 3′ ends. Using 5′‐RACE, we mapped two transcriptional start sites of *PHOX2B‐AS1* to the first intron of *PHOX2B* and an additional start site to its second exon (Fig. 2D). Specifically, the 5′ end variant v1 corresponds to the start site of the transcript annotated in the GenBank database. To determine the 3′ end of *PHOX2B‐AS1*, total RNA from IMR32 cells was reverse transcribed using an oligo‐dT primer with an attached linker sequence. Sequence analysis of PCR‐amplified products identified five distinct 3′ends, three of which were located near the Ensembl‐predicted 3′ end (Fig. 2D).

### Identification of the mouse *Phox2b‐As* transcript

**Fig. 3:**
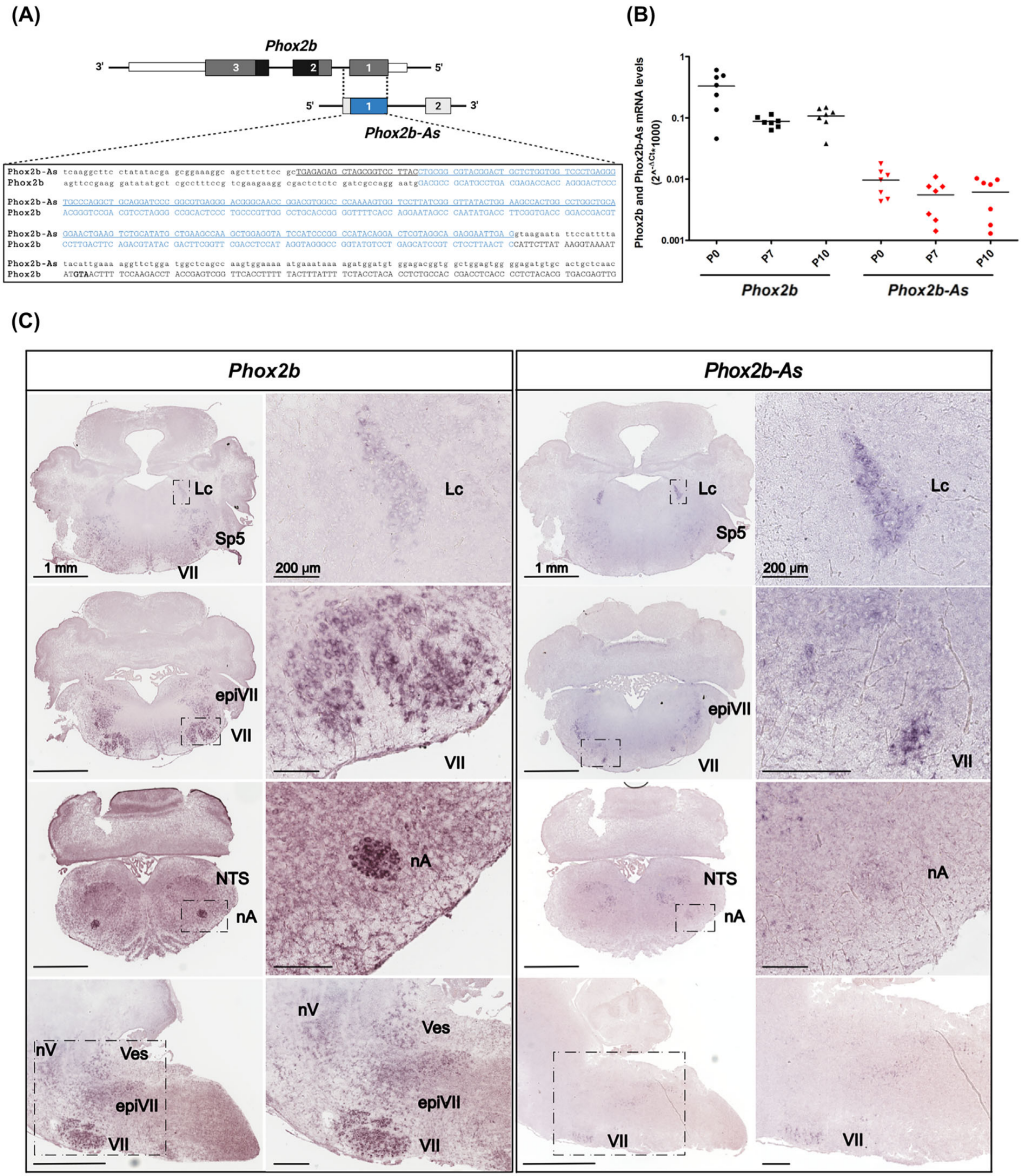
Identification of the mouse *Phox2b‐As* transcript. (A) Schematic representation of *Phox2b* (GenBank reference number: 18935) and *Phox2b‐As* (*Gm33167*; GenBank reference number: 102635966) genes. The sequence corresponds to the region encompassing the first exons of both transcripts, with the two strands manually annotated according to GenBank. The underlined sequence denotes the first exon of *Phox2b‐As*. Uppercase letters represent exonic regions; lowercase letters indicate intronic regions; the putative overlapping region between mature transcripts is highlighted in blue. The Phox2b translation start site (ATG) is shown in bold. (B) qPCR analysis of *Phox2b* and *Phox2b‐As* RNA levels in brains at postnatal days P0, P7, and P10 (*N* = 7/developmental stage). (C) Representative images of ISH (*in situ* hybridization) analysis of *Phox2b* and *Phox2b‐As* expression in P0 mouse brains. *N* = 3 biological replicates per probe. Scale bars = 1 mm and 200 μm (zoomed views). epiVII, epifacial neurons; Lc, locus coeruleus; nA, nucleus ambiguus; NTS, nucleus of solitary tract; nV, trigeminal motor nucleus; Sp5, spinal trigeminal tract; Ves, vestibular neurons; VII, facial motor nucleus.

A predicted antisense gene to mouse *Phox2b* gene has been recently annotated in the GenBank database (*Gm33167*, GenBank reference number: 102635966), whereas no antisense transcript has been annotated yet in the Ensembl database. The *Gm33167* gene encodes two transcript variants (GenBank reference numbers: NR_166598.1 and NR_166599.1), which differ by the presence of an additional exon after exon 1 in transcript variant 1 (Fig. S6). Using 5′ and 3′ RACE, we identified an antisense transcript, which we refer to as the mouse *Phox2b‐As* transcript (Fig. 3). The nucleotide sequence of mouse *Phox2b‐As* is provided in Fig. S7. 5′‐RACE analysis identified a transcription start site (TSS) corresponding to that of the *Gm33167* transcript, along with an alternative one located within the second exon of *phox2b*. This alternative TSS aligns with the 5′ variant 3 of the human *PHOX2B‐AS1* transcript. The mouse *Phox2b‐As* transcript consists of two exons and includes a 217‐nucleotide region complementary to *Phox2b* exon 1 (Fig. 3A). These exons correspond to the first two exons of the transcript variant 1 of gene *Gm33167* (Fig. S8). Although the architectures of *PHOX2B‐AS1* in human and mouse are not entirely conserved, the 217‐bp overlapping region is almost identical, exhibiting 96.3% sequence homology between the two species (Fig. S9). Additionally, the second exon of the mouse *As* shares 99% homology with the extra exon between exons 1 and 2 in human cells (indicated as exon 1a in Fig. 2C and highlighted in green in Fig. S9).

To characterize the expression profile of the murine *Phox2b‐As* transcript, we performed qPCR analysis on RNA extracted from mouse brains at postnatal days P0, P7, and P10. Our results indicate that both *Phox2b* and *Phox2b‐As* are expressed during postnatal stages, with the highest mRNA levels observed at P0, followed by a decrease at P7 and P10 (Fig. 3B), suggesting a concordant expression. *In situ* hybridization (ISH) using strand‐specific probes further confirmed the presence of the *Phox2b‐As* transcript in the mouse brain. Notably, *Phox2b* and *Phox2b‐As* were found to be co‐expressed in several brain regions (Fig. 3C), suggesting a potential regulatory role for *Phox2b‐As* in *Phox2b* expression. In some *Phox2b* positive areas, the weak signal of *Phox2b‐As* confirmed the low abundance of the transcript compared to *Phox2b*.

### Expression profiles of *PHOX2B* and *PHOX2B‐AS1* in human tissues and cell lines

**Fig. 4:**
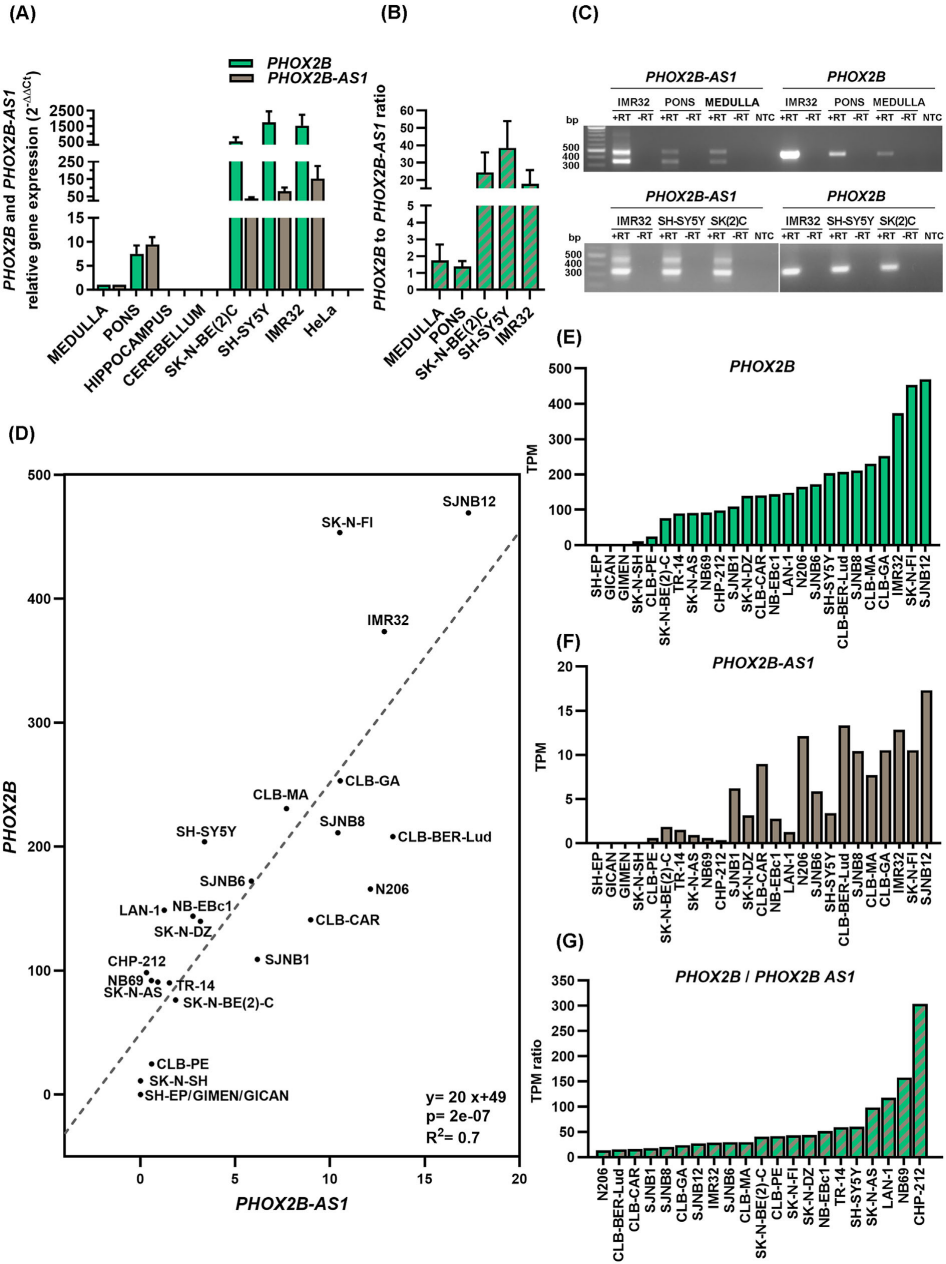
*PHOX2B* and *PHOX2B‐AS1* expression in human tissues and cell lines. (A) qPCR (quantitative PCR) analysis of *PHOX2B* and *PHOX2B‐AS1* expression in various cell lines and human tissues. *N* = (SK‐N‐BE(2)C: 5; SH‐SY5Y: 3; IMR32: 4; medulla, pons, hippocampus, cerebellum, and HeLa: 2) biological replicates, with three technical replicates for each sample. Results are presented as mean ± SD. (B) Expression levels of *PHOX2B* and *PHOX2B‐AS1* transcripts were measured by qPCR in various tissues and neuroblastoma cell lines. The expression of *PHOX2B* was normalized to *PHOX2B‐AS1* in each sample and the bars represent the ratio of *PHOX2B* to *PHOX2B‐AS1* (*PHOX2B/PHOX2B‐AS1*). *N* = (SK‐N‐BE(2)C: 5; SH‐SY5Y: 3; IMR32: 4; medulla and pons: 2) biological replicates, with three technical replicates for each sample. Results are presented as mean ± SD. (C) Conventional PCR analysis confirming the presence of multiple *PHOX2B‐AS1* splice variants in neuroblastoma cell lines and brain tissues (pons and medulla). The image is representative of three independent experiments. (D) Scatterplot showing *PHOX2B* (*y*‐axis) and *PHOX2B‐AS1* (*x*‐axis) TPM (transcripts per million) values across 25 neuroblastoma cell lines. The dotted line represents the linear regression model, where *y* is *PHOX2B* and *x* is *PHOX2B‐AS1*. *N* = 1 biological replicate/cell line. (E, F) Expression levels of *PHOX2B* and *PHOX2B‐AS1* were analyzed using publicly available RNA‐seq data from neuroblastoma cell lines [23]. *N* = 1 biological replicate/cell line. (G) Relative expression of *PHOX2B* normalized to *PHOX2B‐AS1* in each neuroblastoma cell line, highlighting the variability in transcript ratios across different lines. *N* = 1 biological replicate/cell line.

Quantitative PCR analyses, using oligonucleotides able to amplify all splicing variants, showed that *PHOX2B‐AS1* is expressed in different PHOX2B‐positive neuroblastoma cell lines (SK‐N‐BE(2)C, IMR32 and SH‐SY5Y) as well as in human brain areas such as the pons and medulla. In contrast, *PHOX2B‐AS1* was not detected in PHOX2B‐negative cell lines and tissues, suggesting a positive correlation with *PHOX2B* expression (Fig. 4A). Similar to *PHOX2B* [31], *PHOX2B‐AS1* is highly overexpressed in neuroblastoma cells compared to normal tissues. The expression level of *PHOX2B‐AS1* is 38‐ to 153‐fold higher in neuroblastoma cells compared to the medulla and 4‐ to 16‐fold higher than in the pons. The ratio of *PHOX2B* to *PHOX2B‐AS1* (*PHOX2B/PHOX2B‐AS1*) is similar in normal tissues but is at least 10‐fold lower than that of neuroblastoma cells (Fig. 4B). This suggests that *PHOX2B* upregulation in neuroblastoma cells is only partially mirrored by a corresponding increase in *PHOX2B‐AS1* expression.

Conventional PCR analysis further confirmed the presence of different *PHOX2B‐AS1* splicing variants in all tested neuroblastoma cell lines as well as in pons and medulla tissues (Fig. 4C).

We confirmed a significant positive correlation between *PHOX2B‐AS1* and *PHOX2B* expression in neuroblastoma cell lines by analyzing publicly available RNA‐Seq data sets from 25 neuroblastoma cell lines [23] (Fig. 4D). In the three cell lines with neural crest cell (NCC)‐like identity (GIMEN, SH‐EP and GICAN), which are characterized by the absence of *PHOX2B* and noradrenergic marker expression, the *PHOX2B‐AS1* transcript was undetectable (Fig. 4E,F). In contrast, both transcripts were expressed in all other neuroblastoma cell lines, with the exception of the SK‐N‐SH cell line in which *PHOX2B* expression was extremely low and *PHOX2B‐AS1* was absent (Fig. 4E,F). The *PHOX2B*/*PHOX2B‐AS1* expression ratio varied considerably across the neuroblastoma cell lines, with *PHOX2B* transcript levels ranging from 13‐ to 303‐fold higher than those of *PHOX2B‐AS1* (Fig. 4G).

**Fig. 5:**
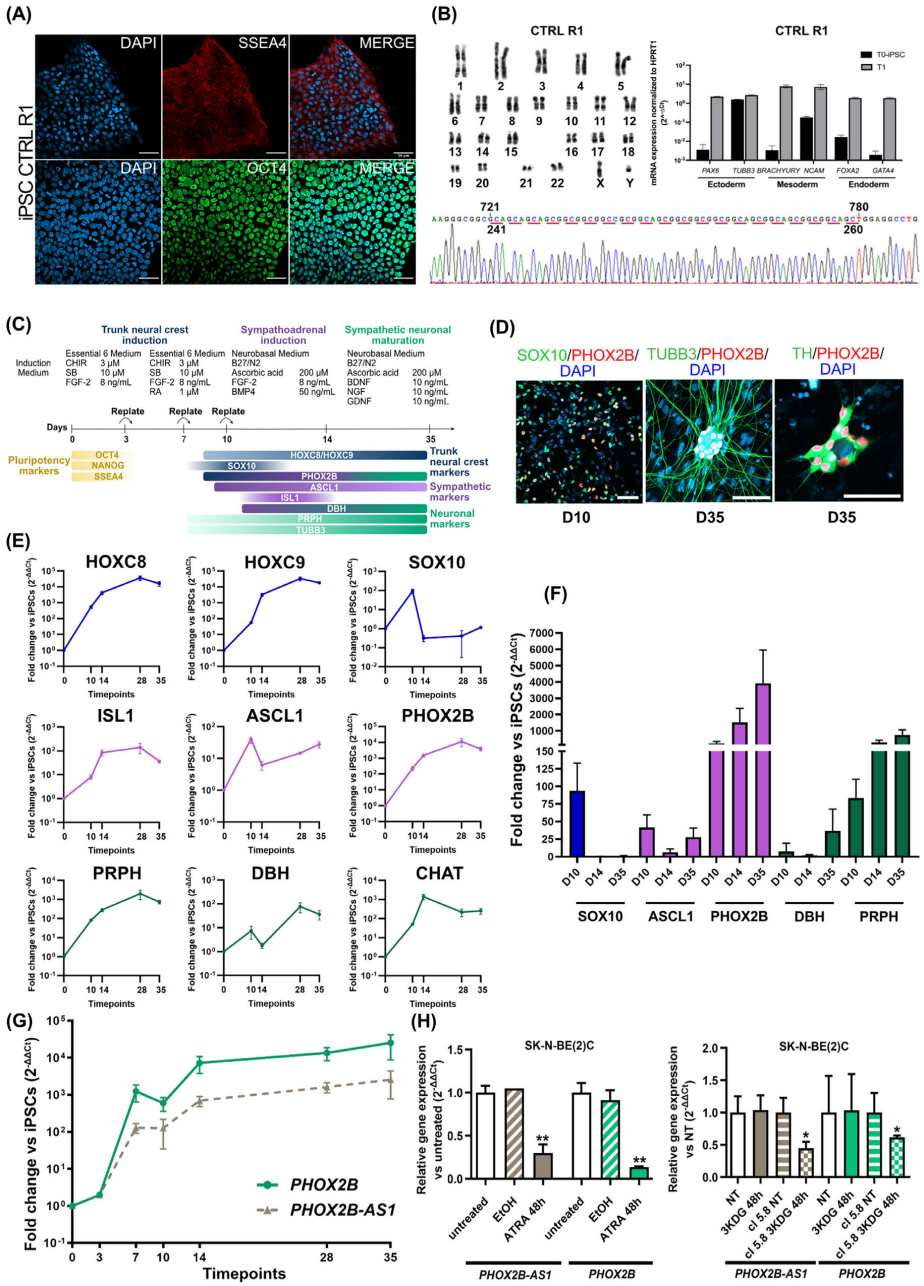
Expression of *PHOX2B* and *PHOX2B‐AS1* during differentiation of iPSC‐derived sympathetic neurons. (A) Immunocytochemistry analysis showing expression of pluripotency markers OCT4 and SSEA4 in the Control #R1 hiPSC (human induced pluripotent stem cell) line. Scale bars = 50 μm. (B) Characterization of the control iPS cell line: on the top left, the karyotype analysis of the Control #R1 iPSCs showing a normal male karyotype (46, XY) with no structural chromosomal abnormalities; on the top right, qPCR analysis of the indicated markers confirmed the ability of the iPSCs to differentiate into ectodermal, mesodermal, and endodermal germ layers. *N* = 2 biological replicates, with three technical replicates for each sample. Results are presented as mean ± SEM; on the bottom, sanger sequencing electropherogram of *PHOX2B* exon 3, demonstrating the presence of the normal 20‐Ala stretch (underlined in red). Numbers indicate the cDNA nucleotide and amino acid numbering corresponding to the 20‐Ala stretch. (C) Schematic overview of the sympathetic neuron differentiation protocol. Key stages, treatments, durations, and marker genes analyzed at each step are indicated. Media components and abbreviations: CHIR (CHIR99021), GSK3β inhibitor and WNT pathway activator; FGF‐2, basic fibroblast growth factor; RA, retinoic acid; BMP4, bone morphogenic protein 4; B27, optimized serum‐free supplement used for the maintenance and maturation of stem cell‐derived neurons; N2 chemically defined, serum‐free supplement based on Bottenstein's *N*‐1 formulation; BDNF, brain‐derived neurotrophic factor; NGF, nerve growth factor; GDNF, glial cell‐derived neurotrophic factor; SB (SB431542), inhibitor of TGFβ receptors ALK4/5/7. (D) Representative immunostaining images of day 10 differentiated cells showing PHOX2B and SOX10 expression, and day 35 iPSC‐derived sympathetic neurons stained for PHOX2B, TUBB3 (β‐III tubulin), and TH (tyrosine hydroxylase). Scale bars = 50 μm. (E) qPCR analysis of the indicated markers was performed at the following time points: 0, 10, 14, 28, and 35 days, to track gene expression changes during *in vitro* differentiation. *N* = (D0, D10, D14: 6; D28: 2; D35: 4) biological replicates, with three technical replicates for each sample. Results are presented as mean ± SEM. (F) qPCR analysis showing temporal expression of stage‐specific marker genes at key differentiation time points. *N* = (D0, D10, D14: 6; D35: 4) biological replicates, with three technical replicates for each sample. Results are presented as mean ± SEM. (G) Temporal expression profiles of *PHOX2B* (solid line) and *PHOX2B‐AS1* (dashed line) during differentiation, measured by qPCR at days 0, 3, 7, 10, 14, 28, and 35. *N* = (D0, D3, D7, D10, D14: 4; D28: 2; D35: 2) biological replicates, with three technical replicates for each sample. Results are presented as mean ± SEM. (H) Expression of *PHOX2B* and *PHOX2B‐AS1* in SK‐N‐BE(2)C cells after treatment with ATRA (all‐trans retinoic acid) (left) and 3‐KDG (3‐keto‐desogestrel) (right). On the left: qPCR analysis of *PHOX2B* and *PHOX2B‐AS1* expression in differentiated SK‐N‐BE(2)C cells treated with all‐trans retinoic acid (ATRA, 10 μm) for 48 h. The bars represent the mean values ± SD of two independent experiments, expressed as fold induction relative to the untreated sample (white bars). Hatched bars represent vehicle‐treated cells (EtOH, ethanol), and green and light brown bars represent ATRA‐treated cells. **P < 0.01 indicates a statistically significant difference in *PHOX2B* and *PHOX2B‐AS1* expression between untreated and ATRA‐treated cells (one‐way ANOVA, post‐Tukey's test). On the right: qPCR analysis of *PHOX2B* and *PHOX2B‐AS1* expression normalized to *GAPDH* in native SK‐N‐BE(2)C (white bars) and Clone 5.8 cells stably expressing human progesterone receptors (hatched bars) treated with 1 nm 3‐KDG (cross‐hatched bars) for 48 h. The bars represent the mean values ± SD of three independent experiments, expressed as fold induction relative to the respective untreated samples (NT) (white and hatched bars). *P < 0.05 indicates statistically significant differences in *PHOX2B* and *PHOX2B‐AS1* expression between treated and untreated SK‐N‐BE(2)C clone 5.8 cells (one‐way ANOVA, Tukey's test).

Since *PHOX2B* plays a critical role in sympathoadrenal development, we next investigated whether *PHOX2B‐AS1* expression parallels that of *PHOX2B* during differentiation. To do this, we employed an *in vitro* differentiation protocol to generate sympathoblasts and sympathetic neurons from human induced pluripotent stem cells (hiPSCs). We established and characterized a control hiPS cell line (Control #R1) (Fig. 5A,B) and successfully differentiated it into sympathetic neurons (Fig. 5C–F). The differentiation protocol, carried out under adherent and chemically defined culture conditions, spanned 35 days (Fig. 5C). Using a modified dual SMAD inhibition strategy, we induced trunk neural crest cells (NCCs) by day 10, as indicated by the expression of *SOX10* (Fig. 5D–F) and the upregulation of *HOXC8* and *HOXC9* (Fig. 5E). Subsequent treatment with BMP4 from day 10 onwards led to increased *PHOX2B* expression and the induction of sympathoadrenal progenitors (SAPs) by day 14. Later‐stage markers, such as *TUBB3*, *PRPH*, and *ISL1*, were robustly upregulated following SAP induction (Fig. 5D–F). By day 35, PHOX2B‐positive cells also expressed TH (Fig. 5D), confirming successful sympathetic neuron differentiation. Finally, we tracked *PHOX2B* and *PHOX2B‐AS1* expression throughout the differentiation process. Both transcripts exhibited a temporally synchronized expression pattern, suggesting coordinated regulation during sympathoadrenal lineage progression (Fig. 5G). The concordant expression of *PHOX2B* and *PHOX2B‐AS1* genes is further supported by the downregulation of the antisense transcript with the differentiating drug all‐trans retinoic acid (ATRA) and the progestin 3‐Keto‐desogestrel (3‐KDG), both previously shown to reduce *PHOX2B* mRNA levels [32, 33] (Fig. 5H).

### PHOX2B regulates *PHOX2B‐AS1* expression

**Fig. 6:**
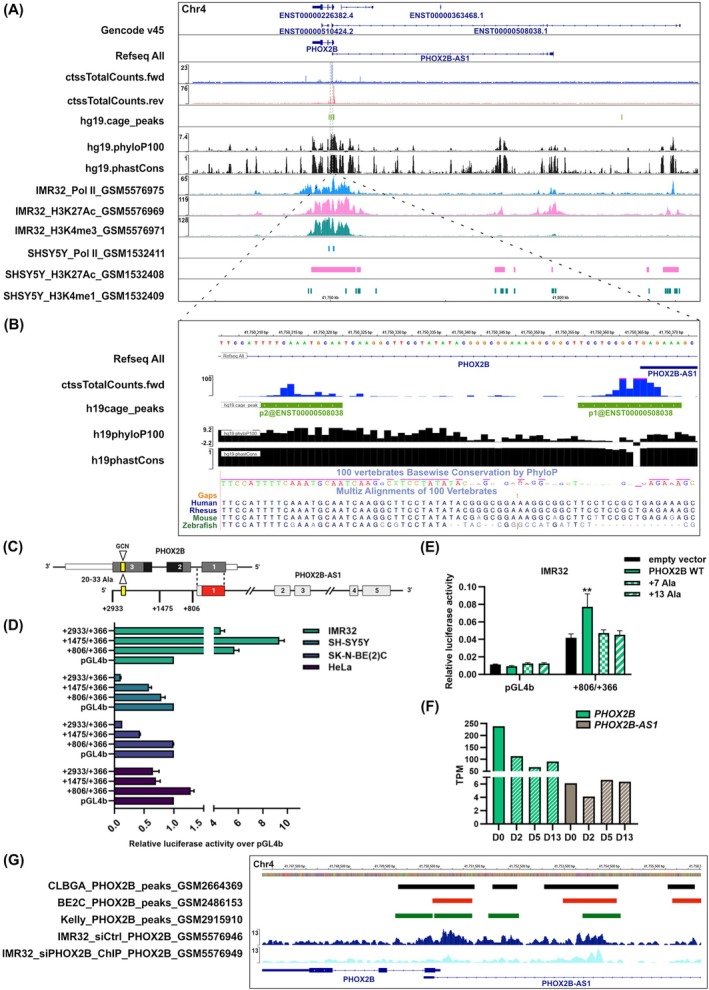
Characterization of the *PHOX2B‐AS1* promoter. (A) Integrated Genome Viewer (IGV) tracks of the *PHOX2B‐AS1* locus. Shown are CAGE (Cap analysis of gene expression)‐seq coverage tracks from FANTOM5 (the blue track represents forward‐strand signal and red peaks indicate minus‐strand signal) across the *PHOX2B/PHOX2B‐AS1* region, PhyloP and phastCons conservation scores across 100 vertebrate species (black), and ChIP (Chromatin immunoprecipitation)‐seq profiles for Pol II, H3K27ac, and H3K4 mono‐ and trimethylation in neuroblastoma cell lines IMR32 and SH‐SY5Y (light blue, pink, and green, respectively). (B) Zoomed‐in view of the putative *PHOX2B‐AS1* regulatory region, highlighting CAGE peaks and sequence conservation obtained from UCSC genome browser (https://genome.ucsc.edu; GRCh37/hg19 assembly) (C) Schematic representation of the *PHOX2B* and *PHOX2B‐AS1* loci. Yellow boxes indicate the GCN repeat region in the *PHOX2B* gene and the corresponding region within the putative *PHOX2B‐AS1* promoter. (D) Luciferase reporter assays showing the transcriptional activity of the full‐length *PHOX2B‐AS1* promoter construct (+2933/+366) and its deletion variants (+1475/+366 and +806/+366) following transfection into IMR32, SH‐SY5Y, SK‐N‐BE(2)C, neuroblastoma cell lines, and HeLa cells. *N* = (IMR32: 9; SH‐SY5Y: 6; SK‐N‐BE(2)C: 4; HeLa: 5) biological replicates. Results are presented as mean ± SEM. (E) Luciferase assays in IMR32 cells showing the transcriptional activity of the *PHOX2B‐AS1* promoter construct (+806/+366) cotransfected with either wild‐type *PHOX2B*, *PHOX2B* + 7 alanine, *PHOX2B* + 13 alanine expression vectors, or empty vector. *N* = (empty vector and wild‐type *PHOX2B*: 18; *PHOX2B* + 7 alanine and *PHOX2B* + 13 alanine expression vectors: 8) biological replicates. Results are presented as mean ± SEM. **Significant differences from the activity of the +806/+366 construct cotransfected with the empty vector (ANOVA, Tukey's test: *P* < 0.01). (F) Expression analysis of *PHOX2B* and *PHOX2B‐AS1* transcripts in the doxycycline‐inducible PHOX2B knockdown model (CLB‐GA‐shPHOX2B). Transcript levels were measured at different time points following doxycycline treatment to assess the effect of *PHOX2B* silencing. *N* = 1 biological replicate. TPM = transcripts per million. (G) Integrated Genome Viewer (IGV) view of peaks in the *PHOX2B* locus region corresponding to the PHOX2B binding sites identified in published ChIP‐Seq experiments [23, 24, 34].

To identify the regulatory region driving *PHOX2B‐AS1* transcription, we analyzed CAGE (Cap analysis of gene expression)‐sequencing profiles from the FANTOM5 consortium (https://fantom.gsc.riken.jp/5/) (Fig. 6A). The blue CAGE track identifies two transcription start sites at 5′ region of *PHOX2B‐AS1*, confirming that it is independently transcribed. The highest peak (indicated as p1@ENST00000508038 in the zoomed view in Fig. 6B) corresponds to the 5′ end variant v1 identified by 5′ RACE experiments in IMR32 (Fig. 2D). Putative TATA and CAAT boxes are located in canonical positions (−25 and −35 bp, respectively). In addition, this region is highly conserved in rhesus and mouse (Fig. 6B). In zebrafish, TATA and CAAT boxes are conserved and the region corresponding to the p1 peak shows a lower degree of conservation. PhyloP100 and phastCons tracks confirm strong conservation of the *PHOX2B/PHOX2B‐AS1* locus across 100 vertebrate species. H3K4me3 profile and Pol II peaks from the neuroblastoma cell lines IMR32 and SH‐SY5Y [34, 35] support the presence of a functional regulatory region upstream of the *PHOX2B‐AS1* TSS (Fig. 6A). Notably, H3K27Ac and H3K4me1 marks indicate that *PHOX2B‐AS1* resides within the super‐enhancer previously reported at the *PHOX2B* locus [23].

To characterize the regulatory region of *PHOX2B‐AS1*, we cloned three genomic fragments corresponding to regions upstream of the antisense transcription start site into a luciferase reporter vector (Fig. 6C) and transfected the constructs into neuroblastoma cell lines (SK‐N‐BE(2)C, IMR32, SH‐SY5Y) and HeLa cells (Fig. 6D). Among these, reporter activity was observed exclusively in IMR32 cells. The shortest fragment (+806 to +366), which includes part of the first intron of *PHOX2B*, induced a sixfold increase in luciferase activity compared to the empty pGL4.10[luc2] vector. Inclusion of the upstream region (+1475 to +806), spanning part of intron 2, exon 2, and part of intron 1 of *PHOX2B*, led to a further increase in luciferase activity. Conversely, reporter constructs containing the region with the GCN repeat tract (+2933 to +366), which is expanded in CCHS patients, exhibited markedly reduced transcriptional activity (Fig. 6D), suggesting the presence of repressive elements within the repeat. Similar decreases in luciferase activity were observed in SK‐N‐BE(2)C and SH‐SY5Y cells. Furthermore, PHOX2B was found to enhance *PHOX2B‐AS1* promoter activity by binding to its proximal promoter region (Fig. 6E). Importantly, PHOX2B proteins carrying PARM mutations were unable to activate luciferase expression, indicating a loss of transcriptional activation capability, as previously reported [36, 37].

We further investigated whether PHOX2B regulates *PHOX2B‐AS1* expression. To this end, we analyzed publicly available RNA‐seq data from a cellular PHOX2B silencing model system using doxycycline‐inducible shRNA construct targeting *PHOX2B* (CLB‐GA‐shPHOX2B) [23]. Along with the expected downregulation of *PHOX2B*, we observed a decrease in *PHOX2B‐AS1* expression after 2 days of doxycycline treatment (Fig. 6F). Interestingly, at later time points (days 5 and 13), *PHOX2B* mRNA levels remained low, but *PHOX2B‐AS1* transcript levels returned to baseline. This suggests that other transcription factors, RNA‐binding proteins, and/or regulatory RNAs may compensate for the absence of PHOX2B to maintain *PHOX2B‐AS1* expression (Fig. 6F). Consistent with this regulatory relationship, PHOX2B chromatin immunoprecipitation sequencing (ChIP‐Seq) data confirmed binding of PHOX2B to a region within the first intron of the *PHOX2B* gene, corresponding to the promoter region of *PHOX2B‐AS1* (Fig. 6G) [23, 24, 38]. ChIP‐seq profiles following PHOX2B silencing confirm the specificity of PHOX2B binding sites [34].

### *PHOX2B‐AS1* positively regulates *PHOX2B* expression at the post‐transcriptional level

Natural antisense transcripts are often involved in the cis‐regulation of neighboring genes. Given that *PHOX2B‐AS1* is transcribed in a 5′head‐to‐head orientation relative to *PHOX2B*, we hypothesized that it may regulate *PHOX2B* expression in *cis*. Supporting this hypothesis, we observed a strong and significant positive correlation between *PHOX2B‐AS1* and *PHOX2B* expression across multiple neuroblastoma cell lines.

**Fig. 7:**
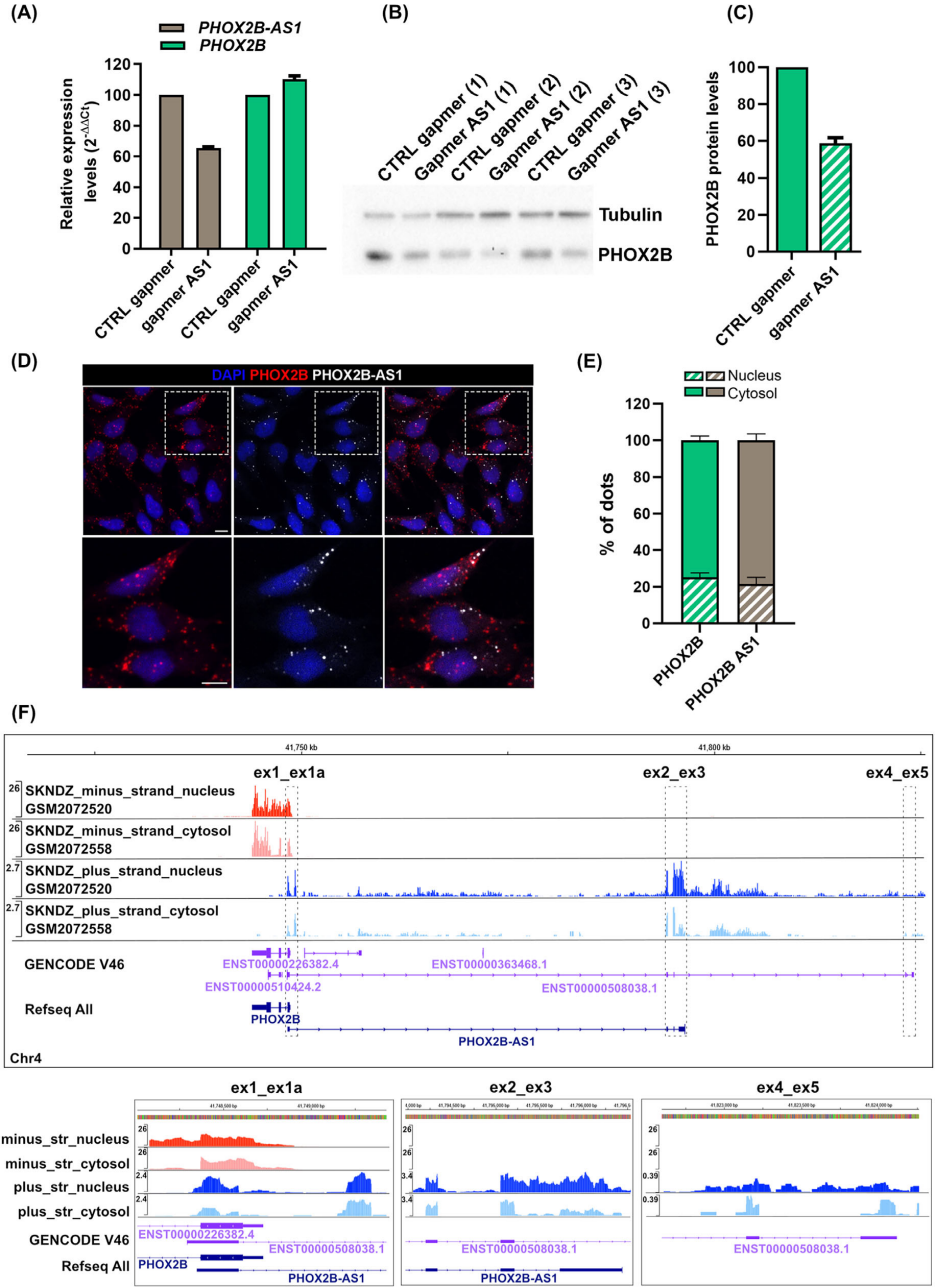
*PHOX2B‐AS1* regulates PHOX2B protein expression. (A) Relative expression levels of *PHOX2B‐AS1* (light brown bars) and *PHOX2B* (green bars) mRNA in IMR32 cells transfected with a pool of GapmeRs targeting *PHOX2B‐AS1*, expressed as a percentage of cells treated with control (CTRL) GapmeR (*N* = 3). Data represent the mean ± SD from three independent experiments. (B) Western blot analysis of PHOX2B protein levels in IMR32 cells after GapmeR‐mediated knockdown of *PHOX2B‐AS1*. Representative blots from three independent experiments are shown. (C) Quantification of PHOX2B protein expression from panel B, normalized to control and expressed as mean ± SD (*N* = 3). (D) RNAscope *in situ* hybridization analysis of *PHOX2B* and *PHOX2B‐AS1* transcripts in IMR32 cells. Nuclei are counterstained with DAPI (blue). Scale bars: 10 μm (main images) and 20 μm (enlargements). (E) Relative quantification of *PHOX2B* (left, green bar) and *PHOX2B‐AS1* (right, light brown bar) transcripts in the nucleus (hatched) and cytoplasm (filled) (*N* = 5 images, total cells analyzed = 149). Results are presented as mean ± SD. (F) RNA‐seq coverage tracks of the *PHOX2B‐AS1* locus following cellular fractionation in SKNDZ neuroblastoma cells. Red and pink indicate minus‐strand reads corresponding to *PHOX2B*; blue and light blue indicate plus‐strand reads corresponding to *PHOX2B‐AS1*. Enlargements of *PHOX2B‐AS1* exonic regions are shown below.

To directly assess whether *PHOX2B‐AS1* regulates *PHOX2B*, we analyzed *PHOX2B* expression following targeted knockdown of *PHOX2B‐AS1*. We designed three LNA‐gapmeR antisense oligonucleotides (ASOs) targeting nonoverlapping regions of the *PHOX2B‐AS1* transcript (Table S1): GapmeR 1 targets exon 5 and GapmeRs 2 and 3 target the region within the first exon of *PHOX2B‐AS1* that overlaps the first intron of *PHOX2B*. Cotransfection of all three GapmeRs into IMR32 neuroblastoma cells led to approximately a 35% reduction in *PHOX2B‐AS1* transcript levels, without a significant effect on *PHOX2B* mRNA levels (Fig. 7A). However, western blot analysis revealed that PHOX2B protein levels were reduced by about 40% following *PHOX2B‐AS1* knockdown (Fig. 7B,C) suggesting that *PHOX2B‐AS1* enhances PHOX2B expression at the post‐transcriptional level, potentially by promoting translation. Given that cytoplasmic NATs are often implicated in translational regulation, we investigated the subcellular localization of *PHOX2B‐AS1* using RNAscope *in situ* hybridization in IMR32 cells. The analysis revealed *PHOX2B‐AS1* transcripts localized in both the nucleus and the cytoplasm, with a predominant cytoplasmic distribution (Fig. 7D,E), supporting a role in post‐transcriptional regulation. In addition, we analyzed publicly available RNA‐seq datasets from the fractionated neuroblastoma cell line SKNDZ to further support our observations (Fig. 7F). The light blue cytosolic track reveals the full 5‐exon transcript, and interestingly the blue nuclear track suggests that *PHOX2B‐AS1* splicing is finely regulated. Junctions between exons 1, 1a, 2, and 3 are efficiently spliced, whereas the inclusion of distal exons 4 and 5 appears less efficient (Fig. 7F, zoomed view).

## Discussion

In this study, we characterized *PHOX2B‐AS1*, a divergent long non‐coding RNA (lncRNA) transcribed on the plus strand, beginning at the first intron of the *PHOX2B* gene and overlapping a portion of its first exon. Our findings indicate that *PHOX2B‐AS1* positively regulates *PHOX2B* translation. We also identified an analogous long antisense RNA in the mouse *Phox2b* locus, where the overlapping region is highly conserved between humans and mice. This suggests that the function of *PHOX2B‐AS1* in promoting PHOX2B translation is likely evolutionarily conserved.

As *PHOX2B‐AS1* is transcribed in the opposite direction to the sense *PHOX2B* gene, it can be classified as a *cis*‐natural antisense transcript (*cis‐*NAT). Cis‐NATs are widespread across eukaryotes, with 20–70% of coding genes associated with such antisense transcripts [3]. These lncRNAs have been shown to regulate gene expression through various mechanisms, including modulation of transcription, mRNA stability, and splicing. However, fewer studies have explored the role of lncRNAs in modulating mRNA translation. The majority of known examples involve inhibition of translation [4, 5, 6]. In contrast, few cis‐NATs have been reported to enhance translation. For instance, in mice, the mRNA translation of *Ubiquitin Carboxy‐Terminal Hydrolase 1* (*Uchl1*) is enhanced by a nuclear cis‐NAT that is exported to the cytoplasm when CAP‐dependent translation is inhibited by rapamycin [39]. In humans, during megakaryocyte terminal differentiation, a primarily nuclear cis‐NAT (*AS‐RBM15*) is exported to the cytoplasm and enhances the translation of the megakaryocyte differentiation regulator RNA‐binding protein 15 (RBM15) [40]. Both *As‐Uchl1* and *AS‐RBM15* are oriented in a 5′ head‐to‐head configuration with the sense coding genes and overlap the 5′ untranslated regions (5′UTRs) of their respective sense mRNAs. In the case of *As‐Uchl1*, this includes the AUG start codon, while for *RBM15*, only the overlapping region with the 5′UTR is sufficient for translation enhancement. In contrast, *As‐Uchl1* has two essential domains, that is, the overlapping region and a nonoverlapping inverted Short Interspersed Nuclear Element B2 (invSINEB2) element, a class of retrotransposable repeat element. However, when we analyzed the *PHOX2B‐AS1* sequence using RepeatMasker (http://www.repeatmasker.org), we did not find an invSINEB2 element, excluding the possibility that *PHOX2B‐AS1* functions as a SINEUP, a class of lncRNA known to enhance translation via SINE elements [41].

Similarly to *As‐Uchl1* and *AS‐RBM15*, *PHOX2B‐AS1* and the *PHOX2B* genes are organized in a 5′ head‐to‐head orientation. However, the overlapping region of *PHOX2B‐AS1* does not include the 5′ UTR or the AUG start codon of *PHOX2B*. In contrast to *As‐Uchl1* and *AS‐RBM15*, the abundance of *PHOX2B‐AS1* in the cytoplasm is not regulated by nucleocytoplasmic shuttling as RNAscope analysis shows that *PHOX2B‐AS1* is enriched in the cytoplasm. Furthermore, *PHOX2B‐AS1*‐mediated enhancement of protein translation is not a response to external stimuli or specific conditions, such as differentiation or stress‐related processes as observed for *As‐Uchl1* and *AS‐RBM15*. This suggests that *PHOX2B‐AS1* regulates the basal translation of *PHOX2B* under normal cellular conditions. Notably, the analysis of RNA‐seq data from the fractionated neuroblastoma cell line SKNDZ reveals that the full 5‐exon transcript is mainly cytoplasmic and that *PHOX2B‐AS1* splicing is finely regulated. In particular, inclusion of distal exons 4 and 5 appears less efficient, raising the possibility that alternative and/or shorter isoforms may exert functional roles in the nucleus.

Our data also show that the *PHOX2B‐AS1* transcript level is transcriptionally regulated by PHOX2B, providing an example of complex reciprocal regulation between sense and antisense genes involving both transcriptional and post‐transcriptional mechanisms. Interestingly, upon *PHOX2B* silencing, *PHOX2B‐AS1* transcript decreased in parallel with reduced PHOX2B protein levels. At later time points, *PHOX2B‐AS1* expression returned to baseline, suggesting that other transcription factors, RNA‐binding proteins, and/or regulatory RNAs may be recruited to restore *PHOX2B‐AS1* levels.

PHOX2B plays crucial roles in embryogenesis, development, and adult physiology [7, 42, 43], and its expression level must be tightly regulated. Consistent with its critical functions, mutations and dysregulation of *PHOX2B* expression contribute to several diseases, including Congenital Central Hypoventilation Syndrome (CCHS), Hirschsprung's disease (HSCR), and neuroblastoma (NB) [15, 20]. Variants of the *PHOX2B* gene include causative mutations in the coding region, particularly in exon 3, as well as common variants found in the general population, located in the coding region, introns, and both the 5′ and 3′ UTR sequences [15]. The most frequent mutations identified in isolated CCHS patients are polyAla repeat expansion mutations, which involve the incorporation of 4–13 alanines into the 20‐residue C‐terminus stretch. In contrast, other types of mutation, including missense, nonsense, and frameshift mutations, are typically associated with the syndromic form of CCHS, as well as sporadic and familiar cases of isolated NB [12, 44, 45].

Our data show that the regulatory region *PHOX2B‐AS1* overlaps with the *PHOX2B* gene. Furthermore, we identified functional binding sites in the first intron of *PHOX2B* and demonstrated that the repeat region containing the GCN tract, which is expanded in CCHS patients, harbors negative transcriptional elements. Numerous studies suggest that NATs contribute to the pathogenesis of several human disorders, including those caused by trinucleotide repeat expansions (reviewed in Refs [46, 47]). Examples include Huntington's disease (HD) and Huntington's disease‐like 2 (HDL2) [48, 49], as well as spinocerebellar ataxia type 7 and 8 [50, 51]. The diseases associated with bidirectional transcription are caused by expansions of polymorphic and unstable CAG/CTG repeats in coding and noncoding regions of genes. These expansions are typically pathogenic when their size exceeds a certain threshold. In contrast, bidirectional transcription has been documented for only one gene, *RUNX2*, which is associated with diseases caused by expansions of stable GCN repeat [52]. Expansion of polyalanine tracts is responsible for at least nine inherited human diseases, including congenital central hypoventilation syndrome, cleidocranial dysplasia, hand‐foot‐genital syndrome, hypopituitarism, blepharophimosis, ptosis epicanthus inversus syndrome, holoprosencephaly, Partington syndrome, synpolydactyly type II, and oculopharyngeal muscular dystrophy. Eight out of these nine polyalanine tract expansion disorders are associated with transcription factors (PHOX2B, RUNX2, HOXA13, SOX3, FOXL2, ZIC2, ARX, and HOXD13), with the only exception being PABPN1, a protein involved in mRNA polyadenylation. An antisense lncRNA has been identified at the *RUNX2* gene locus, but its involvement in the pathogenesis of cleidocranial dysplasia remains unexplored. *PHOX2B‐AS1* provides a novel example of bidirectional transcription associated with a gene locus containing a stable GCN repeat. Interestingly, near the GCN repeats, as observed in other trinucleotide repeat expansion diseases [47], putative binding sites for the transcription regulatory factor CTCF are present. Additionally, analysis of publicly available chromatin immunoprecipitation (ChIP) sequencing data from the ENCODE consortium and the Broad Institute (genome.ucsc.edu/) indicates that CTCF is associated with the region containing the polyalanine repeats in various cell types, including neuroblastoma cell lines. The role of CTCF‐binding sites flanking the GCN repeat and the potential effects of repeat expansions on *PHOX2B‐AS1* transcription warrant further investigation, as this may contribute to the pathogenesis of PHOX2B‐related diseases. Furthermore, our data show that expanded PHOX2B proteins fail to promote *PHOX2B‐AS1* transcription, suggesting that GCN repeat expansion could influence *PHOX2B‐AS1* expression at multiple levels.

In conclusion, our study identifies a novel regulator of PHOX2B and uncovers a complex cross‐regulatory network that contributes to fine‐tuning of *PHOX2B* expression. Further research is required to investigate the contribution of *PHOX2B‐AS1* to the pathogenesis of PHOX2B‐related diseases and its potential as a therapeutic target. From a therapeutic perspective, manipulating *PHOX2B‐AS1* could serve as an effective strategy to indirectly normalize *PHOX2B* gene expression, particularly in neuroblastoma, where downregulation of PHOX2B is desirable. This approach is supported by evidence that *PHOX2B* knockdown in NB cells inhibits both cell growth and tumor growth *in vivo* [23]. Additionally, retinoic acid has been shown to promote differentiation and suppress tumor growth, providing clinical benefits to patients with neuroblastoma. While the molecular mechanisms underlying the efficacy of retinoid treatment are not fully understood, it is clear that retinoic acid decreases *PHOX2B* expression, and PHOX2B downregulation is a key step in its therapeutic action [32, 53]. We also observed that the ratio of *PHOX2B* to *PHOX2B‐AS1* transcript levels varies significantly among neuroblastoma cell lines, with PHOX2B expression being 13‐ to 303‐fold higher than that of *PHOX2B‐AS1*. Given that *PHOX2B‐AS1* mediates PHOX2B translation, it is important to consider that *PHOX2B* mRNA levels may not accurately reflect protein abundance.

In the context of Congenital Central Hypoventilation Syndrome (CCHS), the role of *PHOX2B‐AS1* in disease pathogenesis warrants further investigation. Both *in vivo* and *in vitro* studies suggest that a loss of function mechanism—particularly transcriptional dysregulation—may play a key role in CCHS (reviewed in Refs [54, 55]). Our data indicate that PHOX2B proteins carrying PARM mutations are unable to activate the *PHOX2B‐AS1* promoter, suggesting a potential reduction in *PHOX2B‐AS1* expression in CCHS. This, in turn, may lead to dysregulation of PHOX2B translation. Such a mechanism could help explain the milder respiratory phenotype observed in individuals with whole‐gene deletions and in *Phox2b* haploinsufficient mice [42, 56, 57].

In CCHS, mutated PHOX2B proteins are likely to misfold and aggregate, exerting dominant‐negative or toxic effects, including direct interference with the function of the wild‐type protein [37]. Moreover, these mutant proteins exhibit increased stability [58]. In this context, the further downregulation of *PHOX2B‐AS1* could help mitigate the toxic effects induced by the mutant protein. A nonselective approach reducing not only the mutant PHOX2B levels might therefore be necessary to partially suppress overall *PHOX2B* expression, with the goal of reducing protein aggregation and restoring functional balance. We cannot exclude the possibility that a reduction in wild‐type PHOX2B could be compensated by the recovery of functionality in both the remaining wild‐type and mutant proteins, especially considering that the role of *PHOX2B*—and the impact of its mutations—in adulthood remains largely unexplored.

## Materials and methods

### Chemicals, antibodies, commercial assays, plasmids, and software

All chemicals, antibodies, critical commercial assays, plasmids, and software used in this paper are listed in Table S2.

### Experimental models

#### hPSC line

The human pluripotent stem cell line (R1, male) was generated from fibroblasts of a healthy donor at the Foundation IRCCS Ca’ Granda Ospedale Maggiore Policlinico and was kindly provided by Prof. Corti. The cells were cultured in Essential 8™ Flex medium (Cat#A2858501; Gibco, Waltham, MA, USA) under feeder‐free conditions on Matrigel hESC‐qualified substrate (Cat#CLS354277; Corning, Glendale, AZ, USA). hPSCs were passaged biweekly as clumps using an EDTA (Cat#15575020; Invitrogen, Waltham, MA, USA ) dissociation solution (0.5 mm EDTA/PBS). The cells were maintained at 37 °C and 5% CO_2_ and were routinely tested for mycoplasma contamination. Genomic integrity was periodically assessed through karyotyping.

#### Cell lines

The SK‐N‐BE(2)C (Cat#CRL‐2268; ATCC, Manassas, VA, USA; RRID: CVCL‐0529), IMR32 (Cat#CCL‐127; ATCC; RRID: CVCL‐0346), and SH‐SY5Y (Cat#CRL‐2266; ATCC; RRID: CVCL‐0019) human neuroblastoma cell lines were cultured in RPMI 1640 medium (Cat#15‐041‐CV; Corning), and the HeLa cells (Cat#CCL‐2; ATCC; RRID: CVCL‐0030) were grown in Dulbecco's modified Eagle's medium (DMEM; Cat#15‐013‐CV; Corning). Each medium was supplemented with 10% fetal bovine serum (FBS; Cat#35‐079‐CV; Corning), 100 units·mL^−1^ penicillin, 100 μg·mL^−1^ streptomycin (Cat#30‐002‐CI; Corning), and 2 mm l‐glutamine (Cat#25‐005‐CI; Corning). All cell lines were maintained at 37 °C in a 5% CO_2_ atmosphere and were routinely tested for mycoplasma contamination. The generation of clone 5.8 of the SK‐N‐BE(2)C neuroblastoma cell line stably expressing human progesterone receptors (PGRs) has been previously described [33]. The stable SK‐N‐BE(2)C 5.8 clone was maintained under selection by adding geneticin (G‐418 Sulfate, Cat#10131‐035; Gibco) at a final concentration of 0.4 mg·mL^−1^, with selective medium changes every other day.

#### Mouse strains

ICR mice (ICR/HaJ mice, strain #009122, RRID: IMSR_JAX:009122, The Jackson Laboratory), aged from postnatal day P0 to P10, were used as specified in each figure and figure legend. No significant differences based on sex were observed, and data from both sexes were pooled. The mice were housed in ventilated plastic cages (groups of 4–5 mice/cage), and food and water were provided *ad libitum* and lights were on a standard 12 h light : dark cycle. All procedures followed the ARRIVE guidelines. All animal procedures were carried out in accordance with the Guidelines for the Care and Use of Laboratory Animals and were approved by the Institutional Animal Care and Use Committee (IACUC) at OHSU (protocol # IS1544).

### Reporter and expression plasmids

The MYC‐tagged PHOX2B wild‐type, +7 alanine, and +13 alanine mutant plasmids have been previously described [36, 37]. All oligonucleotides used to generate the reporter constructs are listed in Table S1. PCR amplifications were performed using the GC‐rich PCR system (Cat#04743784001; Roche, Basel, Switzerland). The resulting PCR products were sequenced on both strands and cloned into the pGL4.10[luc2] vector (Cat#E6651; Promega, Madison, WI, USA).

### Transfection with plasmids or gapmeRs

Cells were transiently transfected using lipofection (FUGENE HD, Cat#E2311; Promega). Specifically, 1.6 × 10^5^ SK‐N‐BE(2)C cells, 5 × 10^4^ HeLa cells, 2 × 10^5^ IMR32 cells, and 2 × 10^5^ SH‐SY5Y cells were transfected with 80 fmol of each luciferase reporter constructs. These constructs were combined with 80 fmol of pGL4.74 [hRluc/TK] vector (Cat#E6921; Promega) containing the *Renilla reniformis* luciferase gene under the control of the TK promoter, or 80 fmol of the phRG‐B vector (Cat#E6281; Promega) for SK‐N‐BE(2)C cells. In cotransfection experiments with the PHOX2B expression vector, 80 fmol of expression constructs or the empty vector pcDNA 3.1 Myc‐His were used (Cat#V80020; Invitrogen). Luciferase activity was measured using the Dual‐Luciferase Reporter Assay System (Cat#E1910; Promega) and a GloMax Discovery Luminometer (Promega). All transfections were performed in triplicate, and each construct was tested in at least three independent experiments.

To silence the *PHOX2B‐AS1* transcript, IMR32 cells were transfected with 50 picomoles of each *PHOX2B‐AS1* LNA GapmeR‐1, GapmeR‐2, and GapmeR‐3 (for a total of 150 picomoles, 62.5 nm) (Cat#339511; Qiagen, Hilden, Germany) or 150 picomoles of control GapmeR (Cat#339515; Qiagen) using Lipofectamine™ RNAiMAX Transfection Reagent (Cat#13778075; Invitrogen), following the manufacturer's instructions.

### Cell treatments

Cell treatments with desogestrel were performed by adding 1 nm of the active 3‐keto‐desogestrel metabolite (3‐KDG, Cat#E8797; USBiological, Salem, MA, USA) to the medium for 48 h. All‐trans retinoic acid (ATRA; Cat#R2625; Sigma‐Aldrich, St.Louis, MO, USA), dissolved in 100% ethanol, was added at a final concentration of 10 μm for 48 h, with the medium being changed daily.

### Generation of iPSCs

#### Isolation, culture, and reprogramming of fibroblasts to iPSCs

Skin biopsy was collected from the forearm of a healthy donor using a dermal punch tool (March 2017). The sample was collected under the approval of the ethical committee at the Foundation IRCCS Ca’ Granda Ospedale Maggiore Policlinico and University of Milan (0004520) and with informed consent. The experiments were carried out with the understanding and written consent of the donor, and the study methodologies conformed to the standards set by the Declaration of Helsinki. After mechanical tissue dissociation, the biopsy was cultured for 2 weeks in Dulbecco's modified Eagles medium (DMEM; Cat#11960044; Gibco) supplemented with fetal bovine serum (10%; Gibco), 2 mm l‐glutamine and antibiotics (100 U·mL^−1^ penicillin, 100 μg·mL^−1^ streptomycin) to establish a primary culture of fibroblasts. Fibroblasts were then reprogrammed using the CytoTune™‐iPS 2.0 Sendai Reprogramming Kit (Cat#A16517; Invitrogen), following the manufacturer's instructions. The cells were plated into two wells of a 6‐well plate to achieve 50–60% confluence on the day of transduction (day 0). At day 0, cells from one well were harvested and counted to determine viral volume. Cells from the other well were transduced with the CytoTune™ 2.0 Sendai reprogramming vectors at the appropriate MOI. On day 1, the medium was replaced with fresh complete fibroblast medium to remove the reprogramming vectors. The cells were cultured for six more days, with medium changes every other day. On day 7, the cells were replated onto Matrigel (Cat#CLS354277; Corning)‐coated six‐well plates. From day 8, cells were cultured in Essential 8 medium (Cat#A1517001; Gibco) for 12–20 additional days. Between days 25 and 28, iPSC colonies with embryonic stem cell‐like morphology were selected, picked, and manually transferred onto fresh Matrigel‐coated 12‐well plates for expansion or analyses.

#### Chromosome preparation and karyotype analysis

Karyotype analysis has been performed on the R1‐control iPSCs (between passages 10–15) before carrying out the differentiation step. The cultures were stopped with 70 ng·mL^−1^ colcemid (Sigma‐Aldrich) at 37 °C for 90 min. Chromosome preparations were obtained using standard procedures of the Prenatal and Postnatal Cytogenetics Service of the Department of Medical Biotechnology and Translational Medicine (University of Milan, Italy) and analyzed by QFQ banding (400 bands). Twenty metaphases were examined, and at least five cells were fully karyotyped in accordance with the 2024 International System for Human Cytogenomic Nomenclature (ISCN).

#### Mutation analysis (Sanger analysis)

To amplify *PHOX2B* exon 3, a PCR reaction specific for a GC rich template was set up by using the GC‐Rich PCR System (Cat#12140306001; Roche) with primers 2Bex3‐F: 5′‐TGCTTCACCGTCTCTCCTTCC‐3′ and 2Bex3‐R: 5′‐TACCCGCTCGCCCACTCG‐3′. Reaction mixes were run for 35 cycles at: 95 °C for 1 min, 60 °C for 45 s, and 72 °C for 1 min and 30 s. PCR fragments were purified with the ExoSAP‐IT™ PCR Product Cleanup Reagent (Cat#78201.1.ML; Applied Biosystems, Waltham, MA, USA) by incubating at 37 °C for 40 min and at 80 °C for 15 min and subjected to direct DNA sequencing using the BigDye™ Terminator v1.1 Cycle Sequencing Kit (Cat#4337450; Applied Biosystems) on an ABI 3100 DNA automated sequencer.

Data were analyzed by the sequencher 5.0 software (Gene Codes Corporation, Ann Arbor, MI, USA) and chromatograms visualized by the finch TV 1.4.0 software (Geospiza Inc., Seattle, WA, USA).

### Validation of pluripotency of generated iPSCs

#### Assessment of differentiation potential

The differentiation potential of iPSCs was evaluated by inducing differentiation into all three germ layers using the STEMdiff™ Trilineage Differentiation Kit (Cat #05230; Stem Cell Technologies). Briefly, cells were dissociated into a single‐cell suspension using Gentle Cell Dissociation Reagent (Cat#100‐0485; Stem Cell Technologies, Vancouver, BC, Canada) for 8–10 min at 37 °C, followed by gentle pipetting to ensure complete dissociation. After centrifugation at 300 ***g*** for 5 min, cells were resuspended in E8 Flex medium supplemented with 10 μm rock inhibitor (Y‐27632, Cat#72302; Stem Cell Technologies) to promote survival. Viable cells were manually counted using a hemocytometer and seeded onto matrigel‐coated wells at lineage‐specific densities (200 000 cell·cm^−2^ for ectoderm, 50 000 cell·cm^−2^ for mesoderm, and 100 000 cell·cm^−2^ for endoderm). Cells were cultured in lineage‐specific STEMdiff™ media, with daily medium changes for 5 days (mesoderm and endoderm) or 7 days (ectoderm). Total RNA was extracted using TRIzol™ Reagent (Cat#15596026; Invitrogen) and reverse transcribed into cDNA using the High‐Capacity RNA‐to‐cDNA™ Kit (Cat#4374967; Applied Biosystem). Expression of lineage‐specific markers was analyzed by qRT‐PCR using the 7500 Real‐Time PCR System (Applied Biosystem) and SYBR™ Select Master Mix (Cat#4472908; Thermo Fisher, Waltham, MA, USA). Gene expression levels were normalized to the housekeeping gene *HPRT1* using the 2^−ΔCT^ method. Primer sequences are listed in Table S1.

### Differentiation of iPSCs into sympathetic neurons (symNs)

iPSCs from the healthy donor line CTRL R1 were differentiated into sympathetic neurons using a stepwise protocol designed to mimic *in vivo* sympathetic neuron development (see Fig. 5C for a graphical overview). Trunk neural crest (NC) cells were obtained in a 10‐days long process. On day 0, iPSCs at ~ 90% confluency were dissociated into single cells using Gentle Cell Dissociation Reagent (8–10 min at 37 °C), centrifuged at 300 ***g*** for 4 min, and resuspended in Induction Medium 1 (components are listed in Table S3) supplemented with 10 μm rock inhibitor. Viable cells were counted manually using a hemocytometer and seeded at a density of 7.8 × 10^4^ cells·cm^−2^ (750 000 cells per well in a 6‐well plate) on Matrigel Growth Factor Reduced (GFR, Cat#CLS354230; Corning) matrix‐coated wells. Matrigel‐coated plates were prepared by diluting GFR matrix in DMEM‐F12 (Cat#31330‐038; Gibco) to a final concentration of 0.092 mg·mL^−1^, and 1 mL was added per 6‐well. On day 1, the medium was changed and left until day 3. At day 3, cells were passaged using Gentle Cell Dissociation Reagent for 8–10 min at 37 °C and replated in Trunk Induction medium (components are listed in Table S3) with 10 μm rock inhibitor at a density of 5.2 × 10^4^ cells·cm^−2^ (500 000 cells per 6‐well) on GFR matrix‐coated 6‐well plates. The medium was changed every other day, without rock inhibitor. On day 7, cells were again passaged and seeded at the same density (500 000 cells/6 well). By day 10, when cells reached full confluency, they were dissociated and resuspended in Sympathoadrenal Progenitor (SAP) Induction medium with 10 μm rock inhibitor (see Table S3). Cells were seeded onto GFR‐coated 12‐well plates at densities between 1.3 × 10^5^ and 2.1 × 10^5^ cells·cm^−2^ (500 000–800 000 cells/well). GFR matrix was diluted to 0.15 mg·mL^−1^, and 500 μL were added per well. The medium, without rock inhibitor, was changed the following day and every other day until day 14. On day 14, NC induction concluded, and cells were transitioned to Sympathetic Neuron Maturation Medium (Table S3). The medium was changed every other day until day 35.

### RNA extraction and strand‐specific reverse transcription polymerase chain reaction (SS‐RT‐PCR)

Total RNA was isolated from IMR32 cells using the RNeasy™ Mini kit (Cat#74104; Qiagen) in combination with QIAshredder™ (Cat#79654; Qiagen), following the manufacturer's instructions. To assess the expression of the *PHOX2B‐AS1* transcript, strand‐specific reverse transcription polymerase chain reaction (SS‐RT‐PCR) was performed using one microgram of total RNA and the SuperScript™ III First‐Strand Synthesis System (Cat#18080051; Invitrogen). Reverse transcription was carried out with strand‐specific primers *AS1 RT‐ex4* and *AS1 RT‐ex5*, or with *oligodT* primers. Subsequent PCR amplification was performed using primers *AS1‐F1* and *AS1‐R1* under the following cycling conditions: 95 °C for 45 s, 55 °C for 30 s, and 72 °C for 30 s, for a total of 40 cycles, using GoTaq^®^ G2 Flexi DNA Polymerase (Cat#M7805; Promega). Primer sequences are provided in Table S1, and primer binding sites are illustrated in Fig. 2A.

### Rapid amplification of cDNA ends (RACE) for human *PHOX2B‐AS1* and mouse *Phox2b‐As*

#### 5′ RACE

Ten micrograms of total RNA extracted from IMR32 (human) cells or from N2A (mouse) cells were processed using the FirstChoice^®^ RLM‐RACE Kit (Cat#AM1700; Invitrogen). RNA was first treated with calf intestinal alkaline phosphatase, followed by phenol/chloroform extraction and isopropanol precipitation. Subsequently, RNA was treated with tobacco acid pyrophosphatase to remove 5′ cap structures. At this time, an oligonucleotide adapter was covalently linked to the 5′ end of decapped transcripts. Reverse transcription was performed at 42 °C for 1 h using 2 μL of adapter‐ligated RNA. One microliter of the RT reaction was amplified using *AS1 5′ RACE OUTER* or *mAs 5′ RACE OUTER*, as gene‐specific primers, along with the *5′ RACE OUTER* primer supplied in the kit, using the Expand High Fidelity™ PCR system (Cat#04738250001; Roche). PCR cycling conditions were: 95 °C for 30 s, 60 °C for 30 s, 68 °C for 2 min for 10 cycles, 95 °C for 30 s, 60 °C for 30 s, 68 °C for 2 min + 5 s/cycle for 25 cycles. A nested PCR was then performed using 1 μL of the first‐round PCR product, *AS1 5′ RACE INNER* or *mAs 5′ RACE INNER* as gene‐specific primers, and the *5′ RACE INNER* primer from the kit. PCR cycling conditions were: 95 °C for 30 s, 63 °C for 30 s, 68 °C for 2 min for 10 cycles, 95 °C for 30 s, 63 °C for 30 s, 68 °C for 2 min + 5 s/cycle for 25 cycles. Final PCR products (2 μL) were cloned into the pCRII‐TOPO^®^ TA vector (Cat#45‐0640; Invitrogen). Inserts from 10 positive clones were selected and sequenced on both strands.

#### 3′ RACE

One microgram of total RNA extracted from IMR32 cells was reverse transcribed using the FirstChoice^®^ RLM RACE Kit (Cat#AM1700; Invitrogen). Two microliters of the RT reaction were used for the first‐round PCR with *AS1 3′ RACE OUTER* as the gene‐specific primer and the *3′ RACE OUTER* primer provided in the kit, using the Expand High Fidelity™ PCR system (Cat#04738250001; Roche). PCR cycling conditions were as follows: 95 °C for 30 s, 60 °C for 30 s, 68 °C for 2 min for 10 cycles, 95 °C for 30 s, 60 °C for 30 s, 68 °C for 2 min + 5 s/cycle for 25 cycles. A nested PCR was then performed using 2 μL of the first‐round PCR product with either *AS1 3′ RACE INNER_1* or *AS1 3′ RACE INNER_2* and the *3′ RACE INNER* primer included in the kit. PCR cycling conditions were: 95 °C for 30 s, 63 °C for 30 s, 68 °C for 2 min for 10 cycles, 95 °C for 30 s, 63 °C for 30 s, 68 °C for 2 min + 5 s/cycle for 25 cycles. Two microliters of PCR product were cloned into TOPO‐TA vector (Invitrogen). Inserts from eight positive colonies were sequenced on both strands. For mouse *Phox2b‐As*, one microgram of total RNA from N2A cells was reverse transcribed using the FirstChoice^®^ RLM RACE Kit. Two microliter of the RT reaction was PCR amplified using *mAs 3′ RACE* as the gene‐specific oligonucleotide and the *3′ RACE OUTER* primer from the kit, with the Expand High Fidelity PCR system (Roche). PCR cycling conditions were: 95 °C for 30 s, 65 °C for 30 s, 68 °C for 2 min for 10 cycles, 95 °C for 30 s, 65 °C for 30 s, 68 °C for 2 min + 5 s/cycle for 25 cycles. About 2 μL of PCR product were cloned into the TOPO‐TA vector (Invitrogen). Inserts from five positive colonies were sequenced on both strands.

### Translation and Kozak sequence prediction tools

Putative open reading frames (ORFs) within the *PHOX2B‐AS1* transcript were identified using the ExPASy translate tool (http://web.expasy.org/translate/) [59]. Prediction of Kozak consensus sequences was performed using ATGpr (http://atgpr.dbcls.jp/), a web‐based tool for evaluating translation initiation sites.

### Real‐time quantitative PCR

To assess the expression levels of *PHOX2B* and *PHOX2B‐AS1* transcripts, total RNA was extracted from various cell lines using the RNeasy™ Mini and Micro Kits, along with the QIAshredder™ (Cat#74104, Cat#74004, and Cat#79654; Qiagen), following the manufacturer's protocol. Human RNA from different brain regions was obtained from USB Biological Life Science (Cat#T5595‐7271, Cat#T5595‐7261, Cat#T5595‐7247, Cat#T5595‐7277). Five hundred nanograms of RNA were reverse transcribed using the GoScript™ Reverse Transcriptase kit (Cat#A5001; Promega). Quantitative PCR (qPCR) was then performed on an ABI Prism QuantStudio 5 Thermocycler (Applied Biosystems, CA, USA) to detect *PHOX2B*, *PHOX2B‐AS1*, and *GAPDH* transcripts, and analyzed by the design and analysis v2 software (DA2; Applied Biosystem), according to the MIQE Guidelines [60]. Each reaction (20 μL total volume) included 25 ng of cDNA, Power SYBR^®^ Green PCR Master Mix (Cat#4367659; Applied Biosystems), and specific primers at the following concentrations: 300 nm forward and reverse primers for *PHOX2B* and *PHOX2B‐AS1* (*AS1‐F qPCR/AS1‐R qPCR* and *PHOX2B‐F qPCR/PHOX2B‐R qPCR*); 50 nm forward and 300 nm reverse primers for *GAPDH* (*GAPDH‐F qPCR* and *GAPDH‐R qPCR*). Primer sequences are listed in Table S1. Thermal cycling conditions were as follows: 50 °C for 2 min; 95 °C for 10 min; 40 cycles of 95 °C for 15 s and 60 °C for 1 min. Each reaction was run in triplicate. Expression levels were determined by using the 2^−ΔCT^ and 2^−ΔΔCT^ methods for normalization to *GAPDH* and comparison to the experimental calibrator (set to 1), as indicated in the figure legends. For the quantification of gene expression shown in Fig. 5E,F, TaqMan^®^ Gene Expression Assays (Cat#4331182; Applied Biosystems) were used. Reactions were prepared in a 96‐well format with 10 μL of Gene Expression Master Mix (Cat#4369016; Applied Biosystems), 1 μL of the specific TaqMan assay (listed in Table S4), 1 μL of cDNA, and nuclease‐free water up to 20 μL. The following thermal protocol was applied: 50 °C for 2 min, 95 °C for 10 min, followed by 40 cycles of denaturation at 95 °C for 15 s, annealing and extension at 60 °C for 1 min. All samples were analyzed in triplicate. Expression levels were calculated using the 2^−ΔCT^ and 2^−ΔΔCT^ methods, normalized to *GAPDH*, and expressed relative to the calibrator sample.

### RNA extraction and quantitative real‐time RT‐PCR analysis of mouse medulla

Total RNA at the indicated developmental stages (P0, P7, and P10) was extracted using TRIzol™ reagent (Cat#15596026; Invitrogen) and treated with RNase‐free DNase (Cat#EN0521; Thermo Fisher Scientific). Reverse transcription was performed using SuperScript™ III First‐Strand Synthesis System (Cat#18080051; Invitrogen) following the manufacturer's instructions. Quantitative real‐time PCR was carried out on a LightCycler^®^ 96 System (Roche) using FastStart Essential DNA Green Master (Cat#06402712001; Roche) under standardized cycling conditions. The relative abundance of each target cDNA was determined using a standard curve method and normalized to *18S* RNA levels. Results are presented as fold change relative to *18S*, representing gene‐specific mRNA enrichment. Data were obtained from seven biological replicates per developmental stage (*N* = 7 brains/developmental stage), with each reaction run in duplicate.

### RNA and ChIP sequencing data analysis

RNA sequencing data for neuroblastoma cell lines were obtained from the study of Boeva *et al*. [23], available in the Sequence Read Archive (SRA) under accession number SRP094096. Fastq files for 29 samples (listed in Table S5) were downloaded from SRA by means of the SRA Toolkit. Transcripts per million (TPM) were quantified using Salmon [61] in ‘mapping‐based’ mode. The ‘salmon index’ was built using reference genome ‘GRCh38.p14’ and reference transcriptome ‘v46’.

CAGE profiles were obtained from the FANTOM5 consortium (https://fantom.gsc.riken.jp/5/). PhastCons100 and PhyloP100 tracks were obtained from UCSC genome browser (http://genome.ucsc.edu). Pol II, H3K27ac, H3K4me3, H3K4me1 ChIP‐seq from IMR32 and SH‐SY5Y were retrieved from Refs [34, 35] (GEO IDs: GSE183641 and GSE62725). PHOX2B‐ChIP‐seq data in CLB‐GA cells (GEO ID: GSM2664369) were obtained from Refs [23]; PHOX2B‐ChIP‐seq data in BE2C cells (GEO ID: GSM2486153) and Kelly cells (GEO ID: GSM2915910) were obtained from Ref. [24]; PHOX2B‐ChIP‐seq data from IMR32 transfected with siPHOX2B (GEO ID: GSM5576949) or siCTRL (GEO ID: GSM5576946) were obtained from Ref. [34]. Nuclear (GEO ID: GSM2072520) and cytoplasmic (GEO ID: GSM2072558) RNA‐seq data of SKNDZ cells were obtained from the ENCODE project [62] (https://www.encodeproject.org, with the following IDs: ENCBS707BMT and ENCBS580YYW).

### Immunofluorescence

At the desired time points during differentiation, cells cultured on glass coverslips coated with GFR matrix were processed for immunofluorescence analysis. Cells were washed twice with PBS and fixed with 2% paraformaldehyde (PFA; Cat#157‐8‐100; Electron Microscopy Sciences, Hatfield, PA, USA) and 4% sucrose for 30 min at room temperature. For iPSCs, fixation was performed with 4% PFA and 4% sucrose. After fixation, cells were washed five times with PBS, then incubated twice for 15 min with 0.1 m glycine to quench residual aldehydes. At this point, cells were washed with High Salt‐PBS (HS‐PBS: 500 mm NaCl, 20 mm phosphate buffer pH 7.4) for 5 min and permeabilized in GDB buffer (0.1% gelatin, 20 mm phosphate buffer pH 7.4, 450 mm NaCl, 0.3% Triton X‐100) for 30 min in a humid and dark chamber at room temperature. Primary antibodies were diluted in GDB and incubated overnight at 4 °C. Antibody dilutions were as follows: TH (1:50; Cat#58844; Cell Signaling Technology, Danvers, MA, USA), TUBB3 (1:100; Cat#5568; Cell Signaling Technology), SOX10 (1 : 100; Cat#89356; Cell Signaling Technology), OCT4 (1 : 200; Cat#2840; Cell Signaling Technology), SSEA‐4 (1 : 500; Cat#4755; Cell Signaling Technology), and PHOX2B (1 : 500; Cat#sc‐376993; Santa Cruz Biotechnology, Dallas, TX, USA). Prior to use, primary antibodies were centrifuged at 12 000 ***g*** for 10 min at 4 °C. Following incubation, cells were washed twice for 5 min with HS‐PBS and incubated with the appropriate fluorescently labeled secondary antibodies, diluted and centrifuged in the same manner as the primary antibodies. Incubation was performed for 1 h at room temperature in the dark. Afterward, coverslips were washed twice with HS‐PBS and incubated with DAPI solution (0.1 μL DAPI per 1 mL HS‐PBS) for 5 min at room temperature to counterstain nuclei. Final washes included one with HS‐PBS and one with Low Salt PBS (LS‐PBS: 150 mm NaCl, 10 mm phosphate buffer, pH 7.4), each for 5 min. Coverslips were mounted onto glass slides using ProLong™ Glass Antifade Mountant (Cat#P36982; Invitrogen™). Imaging was performed using a Zeiss LSM800 confocal microscope equipped with a 40× oil‐immersion objective and analyzed with Zeiss zen 3.9 software (Carl Zeiss Microscopy GmbH, Jena, Germany).

### *In situ* hybridization probes

#### mPhox2b‐pBluescriptSK(−)

Mouse *Phox2b* cDNA (NM_008888.4, 234–833 nt, 600 bp probe) was amplified from total RNA of E17.5 brains (pons region) and cloned into the pBluescriptSK(−) vector between the 5′‐EcoRI and 3′‐NotI restriction sites. Following plasmid linearization, corresponding Digoxigenin‐labeled antisense and sense (negative control) RNA probes were generated through *in vitro* transcription under T3 and T7 promoters, respectively.

#### mPhox2b‐As‐pBluescriptSK(−)

Mouse *Phox2b‐As* DNA (Exons 1 and 2547 bp) was amplified using plasmid DNA obtained from the 3′ RACE experiment as a template and cloned into pBluescriptSK(−) between the 5′‐EcoRI and 3′‐NotI restriction sites. After plasmid linearization, Digoxigenin‐labeled antisense and sense (negative control) RNA probes were *in vitro* transcribed under T3 and T7 promoters, respectively. Primer sequences are listed in Table S1.

### *In situ* hybridization analyses

*In situ* hybridization was performed on 18 μm thick frozen brain sections following standard protocol. Digoxigenin‐labeled antisense RNA probes were hybridized to brain sections at 68 °C. After hybridization, sections were washed, incubated with anti‐digoxigenin‐alkaline phosphatase (AP) antibody, and then subjected to colorimetric reaction. Data were collected from three biological replicates per probe/DNA (*N* = 3 brains/probe/DNA). Images were acquired using an Aperio Digital Whole Slide Scanner (Leica Biosystems, Nussloch, Germany) to ensure consistent background uniformity across different experiments.

### RNAScope analysis

The localization of *PHOX2B mRNA* and *PHOX2B‐AS1* was analyzed by RNAscope, performed following the manufacturer's protocols for ‘RNAscope Assay for Adherent Cells Cultured on Coverslips’ (MK 50‐012; ACDBio, Newark, CA, USA) and ‘RNAscope Multiplex Fluorescent v2 Assay’ (UM 313100; ACDBio), using the RNAscope Multiplex Fluorescent Reagent Kit V2 (Cat. No. 323100; ACD). Cell Preparation: IMR32 cells were plated on poly‐l‐lysine‐coated 12 mm cover glasses (Cat#P2636; Sigma‐Aldrich) at a density of 1 × 10^5^ cells per well. To prepare the cover glasses, 80 μL of poly‐l‐lysine were added, and the glasses were incubated at 37 °C overnight. Excess poly‐l‐lysine was removed, and the cover glasses were allowed to dry for 1 h. The cell suspension was then added, allowing the cells to settle for 10–12 min, after which the appropriate volume of cell culture medium was added.

Cell fixation and permeabilization: When cells reached 50–70% confluence, they were washed twice with PBS, fixed in 4% PFA for 30 min at room temperature, and washed three times with PBS. The cells were dehydrated using an ethanol series (50%, 70%, 100%) for 1 min each at room temperature. The cover glasses were immobilized on glass slides with small drops of nail polish and circled using an Immedge hydrophobic barrier pen (Cat#310018; ACD). Cells were then rehydrated (100%, 70%, 50% ethanol), washed once, and permeabilized in 1× PBS + 0.1% Tween 20 (Cat#28320; Thermo Fisher Scientific) for 10 min at room temperature, followed by two washes. The slides were treated with hydrogen peroxide (2 drops, 10 min at room temperature), then washed twice. Protease treatment was performed with 60 μL of protease III (diluted 1 : 15 in 1× PBS) for 10 min at room temperature, followed by double washing. Probe hybridization: The slides were incubated with 120 μL of positive and negative control probes (Cat#320861 and Cat#320871; ACD), as well as probes for *PHOX2B* (Hs‐PHOX2B‐C2, Cat#567701‐C2; ACD) and *PHOX2B‐AS1* (Hs‐PHOX2B‐AS1‐01, Cat#1245601‐C1; ACD) for 2 h at 40 °C. Amplification and fluorescent detection: Slides were washed twice with Wash Buffer 1× (prepared from RNAscope Wash Buffer 50×, Cat#310091; ACD), and target‐binding amplification was performed by incubating with RNAscope Multiplex FL v2 Amp1 (Cat#323110; ACD) for 30 min at 40 °C, followed by two washes with Wash Buffer 1×. This procedure was repeated with Amp2 and Amp3, with Amp3 requiring 15 min of incubation.

Afterward, the slides were incubated with TSA Vivid fluorophores 570 and 650 (diluted 1 : 1500 in RNAscope Multiplex TSA Buffer, Cat#322809; ACD) to detect the probes. Slides were treated with RNAscope Multiplex FL v2 HRP‐C1/C2 (Cat#323110; ACD) for 15 min at 40 °C, washed twice, incubated with the fluorophore solution for 30 min at 40 °C, washed twice, and then treated with RNAscope Multiplex FL v2 HRP blocker (Cat#323110; ACD) for 15 min at 40 °C. This process was repeated for each channel involved in the experiment. Mounting and imaging: Slides were incubated with DAPI (4 drops, 30 s) for nuclear counterstaining, followed by mounting with ProLong™ Glass Antifade Mountant (Cat#P36982; Invitrogen™). The slides were covered with glass coverslips, dried for 30 min in the dark, and stored at 2–8 °C. Images were acquired using a Leica Stellaris 8 DLS confocal microscope (40× objective) and analyzed with las x Office software (Leica Microsystems, Wetzlar, Germany). For quantification, dots from five images (149 cells in total) were manually counted in the nucleus and cytoplasm using imagej (NIH, Bethesda, MD, USA). The percentage in each compartment was calculated relative to the total number of dots.

### Protein extraction and western blotting

IMR32 cells were transfected with 50 picomoles of *PHOX2B‐AS1* LNA GapmeR‐1, ‐2, and ‐3 (for a total of 150 picomoles, 62.5 nm) or 150 picomoles of a control GapmeR. At 48‐h post‐transfection, cells were collected by centrifugation and lysed in concentrated 3× SDS lysis buffer (6% SDS, 1% NP‐40, 60 mm Tris/HCl, pH 8.5, 10% (vol/vol) 2‐mercaptoethanol, 25% (vol/vol) glycerol, 0.3% (w/vol) bromophenol blue) by incubation at 37 °C for 30 min with continuous agitation. Samples were then diluted to 1× SDS lysis buffer and boiled for 5 min at 100 °C. Viscosity was reduced by passing the lysates through a 22‐gauge syringe needle. Equal aliquots of the lysates were analyzed by western blotting. PHOX2B protein was detected using a primary mouse anti‐PHOX2B antibody (1 : 10 000; Cat#sc‐376997X; Santa Cruz Biotechnology), and β‐tubulin by mouse anti‐β tubulin antibody (1 : 1000; Cat#86298; Cell Signaling Technology). After appropriate washes, protein bands were visualized using LiteAblot EXTEND (Cat#EMP013001; Euroclone, Pero, Italy), with secondary antibodies conjugated to horseradish peroxidase, and images acquired by using the ChemiDoc Imaging System (Bio‐Rad).

Densitometric analysis of the western blot signals was performed using imagelab 6.0 (Bio‐Rad, Hercules, CA, USA). The results are presented as the mean ± standard deviation (SD) of three independent experiments.

### Quantification and statistical analysis

Details of the statistical analyses for the experiments are provided in the figure legends. Data are presented as the mean ± SD or mean ± SEM. All statistical analyses were performed using graphpad prism (GraphPad Software Inc., San Diego, CA, USA) 10.3.0. One‐way ANOVA with Tukey's tests to compare multiple groups. Statistical differences were considered significant with *P* < 0.05 as indicated in figure legends.

## Conflict of interest

The authors declare no conflict of interest.

## Author contributions

SDL and RB contributed to conceptualization; SDL, ALCG, MB, FCh, FCa, PP, IC, PR, SC, VT, and RB contributed to investigation; SDL and EM contributed to formal analysis; SC, PP, and EM contributed to resources; SDL, MB, and FCh contributed to writing—original draft; SDL, ALCG, MB, FCh, FCa, EM, PP, IC, PR, SC, RB, and DF contributed to writing—review and editing; SDL, ALCG, FCh, and MB contributed to visualization; SDL, RB, and DF contributed to project administration; SDL, RB, and DF contributed to supervision; DF contributed to funding acquisition. All authors have read and agreed to the published version of the manuscript.

## Supporting information


**Fig. S1.** Summary and sequence analysis of the *PHOX2B‐AS1* transcripts annotated in Ensembl and NCBI databases, related to Fig. 1.
**Fig. S2.** UCSC genome browser visualization of *PHOX2B‐AS1* transcripts annotated in the Ensembl database (release 113, October 2024), related to Fig. 1.
**Fig. S3.** Summary of *PHOX2B‐AS1* transcripts annotated in the Ensembl database (release 113, October 2024), related to Fig. 1.
**Fig. S4.** Predicted open reading frames and ATGpr analysis of *PHOX2B‐AS1*, related to Fig. 1.
**Fig. S5.** Sequences of human *PHOX2B‐AS1* splicing variants based on PCR sequencing, related to Fig. 2.
**Fig. S6.** Genomic location of the *Phox2b* and *Gm33167* transcripts as annotated in the UCSC Genome Browser, related to Fig. 3.
**Fig. S7.** Sequence of mouse *Phox2b‐As* identified by 5′ and 3′ RACE, related to Fig. 3.
**Fig. S8.** Alignment of *Phox2b‐As* with *Gm33167* transcript variant 1, related to Fig. 3.
**Fig. S9.** Alignment of mouse *Phox2b‐As* with human *PHOX2B‐AS1 (1a)*, related to Fig. 3.
**Table S1.** List and sequences of primers and gapmeRs used for experiments.
**Table S2.** Chemicals, antibodies, commercial assays, plasmids and software.
**Table S3.** Differentiation media.
**Table S4.** List of TaqMan assays used in this study.

**Table S5.** RNAseqIDs.

## Data Availability

The data that support the findings of this study are available from the corresponding authors (simona.dilascio@unimi.it; diego.fornasari@unimi.it) upon reasonable request.
