# Supplementary material for: Regulation of PHOX2B gene expression by the long non‐coding natural antisense RNA PHOX2B‐AS1
Source: FEBS J. 2026 Jan 24;293(12):3502–27. doi: 10.1111/febs.70410 (PMC13278362; doi:10.1111/febs.70410)
Supplement: Supplementary file 1 — Fig. S1. Summary and sequence analysis of the PHOX2B‐AS1 transcripts annotated in Ensembl and NCBI databases, related to Fig. 1. Fig. S2. UCSC genome browser visualization of PHOX2B‐AS1 transcripts annotated in the Ensembl database (release 113, October 2024), related to Fig. 1. Fig. S3. Summary of PHOX2B‐AS1 transcripts annotated in the Ensembl database (release 113, October 2024), related to Fig. 1. Fig. S4. Predicted open reading frames and ATGpr analysis of PHOX2B‐AS1, related to Fig. 1. Fig. S5. Sequences of human PHOX2B‐AS1 splicing variants based on PCR sequencing, related to Fig. 2. Fig. S6. Genomic location of the Phox2b and Gm33167 transcripts as annotated in the UCSC Genome Browser, related to Fig. 3. Fig. S7. Sequence of mouse Phox2b‐As identified by 5′ and 3′ RACE, related to Fig. 3. Fig. S8. Alignment of Phox2b‐As with Gm33167 transcript variant 1, related to Fig. 3. Fig. S9. Alignment of mouse Phox2b‐As with human PHOX2B‐AS1 (1a), related to Fig. 3. Table S1. List and sequences of primers and gapmeRs used for experiments. Table S2. Chemicals, antibodies, commercial assays, plasmids and software. Table S3. Differentiation media. Table S4. List of TaqMan assays used in this study. [file FEBS-293-3502-s001.pdf]

SUPPORTING INFORMATION

Regulation of *PHOX2B* gene expression by the long non-coding natural antisense RNA *PHOX2B-AS1*

Simona Di Lascio[1,*], Ana Lucia Cuadros Gamboa[1,10], Martina Bertocchi[1], Filippo Chiesa[1], Francesca Cargnin[2], Ettore Mosca[3], Paride Pelucchi[3], Viviana Tritto[1], Stefania Corti[4,5,6], Isabella Ceccherini[7], Paola Riva[1], Roberta Benfante[1,8,9,#] and Diego Fornasari[1,#,*]

[1]Department of Medical Biotechnology and Translational Medicine, Università degli Studi di Milano, Milan, Italy
[2]Papé Family Pediatric Research Institute, Department of Pediatrics, Oregon Health & Science University, Portland, OR 97239, USA
[3]Institute of Biomedical Technologies, National Research Council, Segrate (Milan), Italy
[4]Department of Pathophysiology and Transplantation, Dino Ferrari Center, University of Milan, Milan, Italy
[5]Neurology Unit, Foundation IRCCS Ca' Granda Ospedale Maggiore Policlinico, Milan, Italy.
[6]Center for Preclinical Research, Foundation IRCCS Ca' Granda Ospedale Maggiore Policlinico, Milan, Italy
[7]UOSD Aggregation Area of Research Laboratories, IRCCS Istituto Giannina Gaslini, Genoa, Italy
[8]CNR- Neuroscience Institute, Vedano al Lambro (MB), Italy
[9]NeuroMi - Milan Center for Neuroscience, University of Milano Bicocca, Milan, Italy
[10]Present address: Human Technopole, Viale Rita Levi-Montalcini 1, 20157, Milan, Italy
#Senior author

*Correspondence to:
Simona Di Lascio, PhD
Dept. of Medical Biotechnology and Translational Medicine (BIOMETRA), University of Milan
c/o LITA - Fratelli Cervi 93, 20054 - Segrate, (MI) – Italy
Tel: 0039 0250330465
E-mail: simona.dilascio@unimi.it

Prof. Diego Fornasari,
Dept. of Medical Biotechnology and Translational Medicine (BIOMETRA), University of Milan
Via Vanvitelli 32, 20129 Milano, Italy
Tel: 0039 0250316960
E-mail: diego.fornasari@unimi.it

Running Title

PHOX2B-AS1 REGULATES *PHOX2B* GENE EXPRESSION

Figure S1. Summary and sequence analysis of the *PHOX2B-AS1* transcripts annotated in Ensembl and NCBI databases, related to Figure 1

Clustal Omega multiple sequence alignment of *PHOX2B-AS1* transcripts from Ensembl and NCBI databases shows conserved regions across annotations. * indicate conserved nucleotides. Exons are highlighted by alternating black and blue nucleotide sequence.

Database	Gene ID	Gene name (Symbol)	Transcript ID	Transcript name	Length (nt)	N° of exons	Exons lenght (nt)
**Ensembl**	ENSG00000250467	PHOX2B antisense RNA 1 (PHOX2B-AS1)	ENST00000508038.1	PHOX2B-AS1-201	907	5	Exon 1: 294<br>Exon 2: 135<br>Exon 3: 159<br>Exon 4: 83<br>Exon 5: 236
**NCBI**	105374425	PHOX2B antisense RNA 1 (PHOX2B-AS1)	NR_187403.1	PHOX2B-AS1	1240	4	Exon 1: 238<br>Exon 2: 135<br>Exon 3: 159<br>Exon 4: 708

```
CLUSTAL O(1.2.4) multiple sequence alignment


ENST00000508038.1      TTTTCAAATGCAATCAAGGCTTCCTATATACGGGCGGAAAGGCGGCTTCCTCCGCTGAGA  60
NR_187403.1            --------------------------------------------------------GAGA  4
                                                                               ****

ENST00000508038.1      AAGCTGAAGGTCCTTACCTGCGGCGTACGGACTGCTCTGGTGGTCCCTGAGGGTGCCCAG  120
NR_187403.1            AAGCTGAAGGTCCTTACCTGCGGCGTACGGACTGCTCTGGTGGTCCCTGAGGGTGCCCAG  64
                       ************************************************************

ENST00000508038.1      GCTGCAGGATCCCGGCGTGAGGGAAGGGCAGCCGGACGTGGCCCCAAAAGTGGTCCTTAT  180
NR_187403.1            GCTGCAGGATCCCGGCGTGAGGGAAGGGCAGCCGGACGTGGCCCCAAAAGTGGTCCTTAT  124
                       ************************************************************

ENST00000508038.1      CGGGTTATACTGGAAGCCACTGGCCTGGCTGCAGGAACTGAAGTCAGCATAGGCTGAAGC  240
NR_187403.1            CGGGTTATACTGGAAGCCACTGGCCTGGCTGCAGGAACTGAAGTCAGCATAGGCTGAAGC  184
                       ************************************************************

ENST00000508038.1      CAGGCTCGAGGTGTCCATCCCAGCCATACAGGACTCGTAGGCAGAGGAATTGAGGGTTCT  300
NR_187403.1            CAGGCTCGAGGTGTCCATCCCAGCCATACAGGACTCGTAGGCAGAGGAATTGAGGGTTCT  244
                       ************************************************************

ENST00000508038.1      CACAACCAATTGAAAGAGAATAAAACATTCTCTTGCTTCATAACCAAAGTGCTGCTCACA  360
NR_187403.1            CACAACCAATTGAAAGAGAATAAAACATTCTCTTGCTTCATAACCAAAGTGCTGCTCACA  304
                       ************************************************************

ENST00000508038.1      CTTACAGCAACCTTTGGAAGGGGAATCCTGGCTATTGATGTCCCTGGAAAGAGAGTGGTA  420
NR_187403.1            CTTACAGCAACCTTTGGAAGGGGAATCCTGGCTATTGATGTCCCTGGAAAGAGAGTGGTA  364
                       ************************************************************

ENST00000508038.1      GAAGAAAAGAAATTGAAGACATGGAAGAAAGATGAAAACGCTGGCTACTAAATACCTATG  480
NR_187403.1            GAAGAAAAGAAATTGAAGACATGGAAGAAAGATGAAAACGCTGGCTACTAAATACCTATG  424
                       ************************************************************

ENST00000508038.1      TGTGGTTACTGAGTGTGGTATACTTGGACACCGAGCTCCCCCACCCATCAGATGTCACAA  540
NR_187403.1            TGTGGTTACTGAGTGTGGTATACTTGGACACCGAGCTCCCCCACCCATCAGATGTCACAA  484
                       ************************************************************

ENST00000508038.1      AGAGTCCTCTTACTCTTGGATAATTTCAAATCAAACTGACCTCCCCAGATATTGAGAAGC  600
NR_187403.1            AGAGTCCTCTTACTCTTGGATAATTTCAAATCAAACTGACCTCCCCAGGCCAAGCTGAAG  544
                       ************************************************     *   *

ENST00000508038.1      AAAATAATCAACAACAACAAAAC--------CTGCCTTGTAAATATGATTTACATTGATT 652
NR_187403.1            GTATTGAGAAAATATCCAGATGACACAGAAACTGGAATGCTCAGAGAC---TGATGAACA 601
                         * * *  **  *     *           ***   **   * *        **  *
```

```
ENST00000508038.1      ATGTGGGGATGTTCTGAAGG------------GAGAAGAAAGGAGCTGTCACCAACTCAA 700
NR_187403.1            AAGGGAGAATTTTGCTCCTGTTGCTTATTCCTGGGACACCTCTAGCTGGGAATACATATC 661
                       * * * * ** **      *            * **       *****  *  *  *

ENST00000508038.1      AGCAACATGAGAGCAAGAATCATGCTAATTTTGCCGTCTGCCTCTCCTATGTTCTTGGTG 760
NR_187403.1            CCAGGGCTGTTAGTAGTTTTAACCTTTCTTTGGCCACTTGATGGTCGAACCATTCTAGTA 721
                              **  ** *    * *   *  *** ***   **    **  *   *  * **

ENST00000508038.1      CCAGTCTGTAGTTGAGG---------------------TTCATTTACCTGAATGAGTGA 799
NR_187403.1            -CATCCCTGTGCCTAAGGGCTCTCAGAATACTCTGGGAATACAAGTTCGTGCTCAAGGTC 780
                        **   ****   * ***                     * **  * * **    **

ENST00000508038.1      CTAAAGCTTTATAACTTGAACACTTTGAATACATAGATCTAGCATAAATAAGCCTATTAA 859
NR_187403.1            CAACAGCTGTCAGAGCAGATGGCTGGGGA----------TTTGTTTACTGTGGTCCTAAG 830
                       * * **** *   *   **   **  * *          *    * * *  *    * *

ENST00000508038.1      TGGCAACTTTGACCTGTCAAGCAATAACCA-------AATGATTTGTGTGCCTGC----- 907
NR_187403.1            TATTACCCTTTAGCTCTAAGGCACTGGGAACAATTTGACAAATTTGATTAACTGAGAACA 890
                       *   * * ** * ** * * *** *    *       *   *****  *  ***

ENST00000508038.1      ------------------------------------------------------------ 907
NR_187403.1            ATATGATGCTTGCCTGGAAAGCTCACTCCACACTTTCACAGAGTTCCTTATGTTCTTTTC 950

ENST00000508038.1      ------------------------------------------------------------ 907
NR_187403.1            ATGGTCTGAGGATACAGGATGATATTAGCTACCACCTCAGTCCAATGAAACTATCAGAGA 1010

ENST00000508038.1      ------------------------------------------------------------ 907
NR_187403.1            AAACAAAAAAGTCACCCTTGGCTACCCCTTTTGTTTTACAACTGAATGTTTGTGGTTCAC 1070

ENST00000508038.1      ------------------------------------------------------------ 907
NR_187403.1            ATGTACAGCCAGCTGTTCTATCAGAGATGCTCAGAGCATACCTAGTCCTGCCTTTAAGAA 1130

ENST00000508038.1      ------------------------------------------------------------ 907
NR_187403.1            AAAGAAATGTGACTTCATGTTCCATGGCACTCTATAGCTTATTTGTCAATTACAATCTGT 1190

ENST00000508038.1      --------------------------------------------------           907
NR_187403.1            GTCTGCTTTGTGAAACTAAAAATAAATAGTTAATCTGCTGGAAAATCTTA           1240
```

Figure S2. UCSC genome browser visualization of *PHOX2B-AS1* transcripts annotated in the Ensembl database (release 113, October 2024), related to Figure 1

The PHOX2B-AS1/NR_187403.1 transcript annotated in the NCBI database is displayed in blue. The PHOX2B-AS1/ENST00000508038.1 transcript (GENCODE, v46) is shown in purple. *PHOX2B-AS1* transcripts from Ensembl database (release 113) are depicted in green. Panels A, B and C represent magnified views of the regions highlighted in the top panel. Asterisks indicate the ENST00000508038.2 (*PHOX2B-AS1-201*) and ENST00000819364.1 (*PHOX2B-AS1-214*) transcripts, which show 93.2% and 100% similarity to previously annotated transcripts in Ensembl and NCBI databases, respectively.

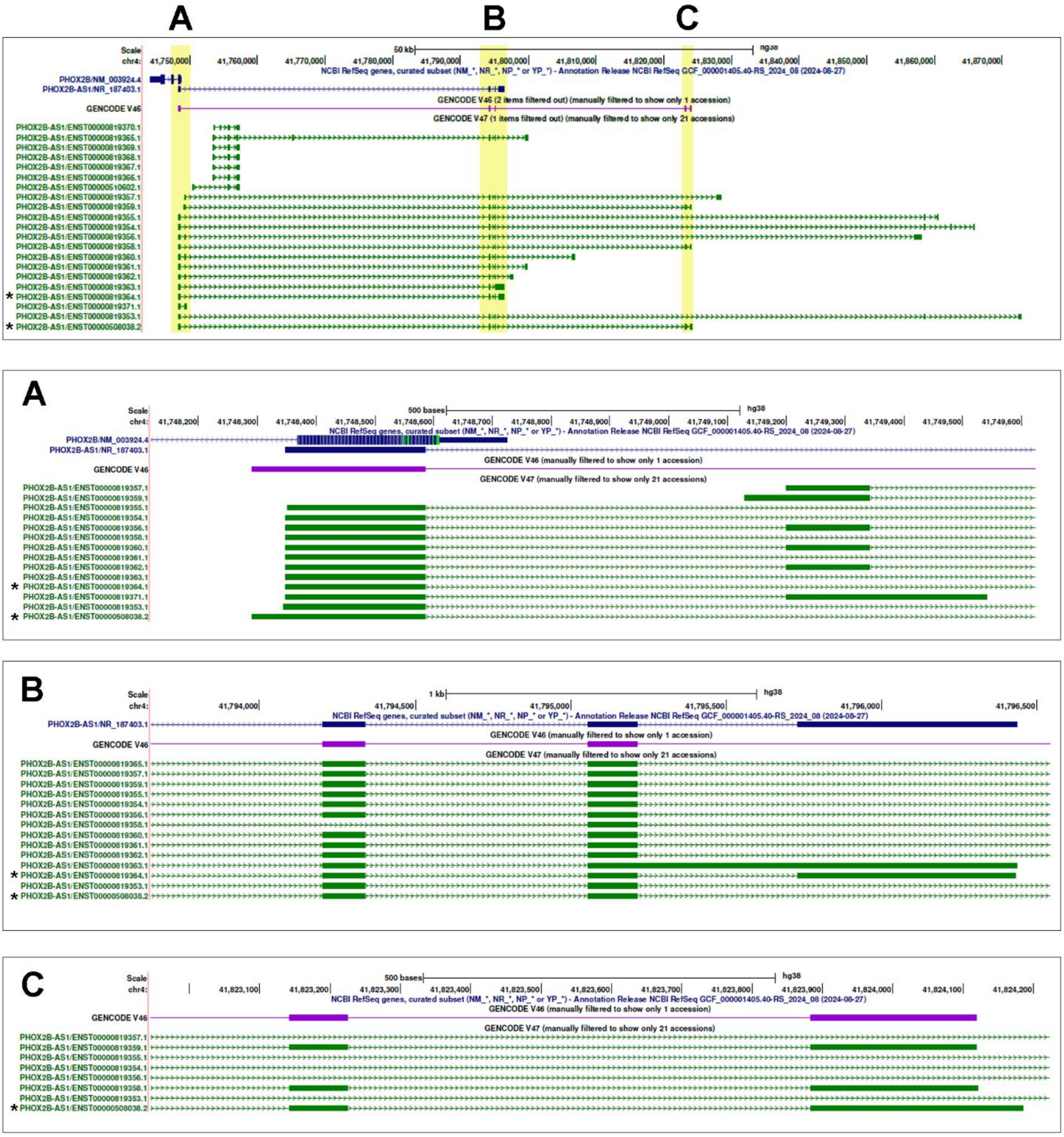

Figure S3. Summary of *PHOX2B-AS1* transcripts annotated in the Ensembl database (release 113, October 2024), related to Figure 1

Transcript variants ENST00000508038.2 (*PHOX2B-AS1-201*) and ENST00000819364.1 (*PHOX2B-AS1-214*), which share 93.2% and 100% sequence similarity with previously annotated transcripts in the Ensembl and NCBI databases, are shown in purple and blue, respectively. Transcript variants with high sequence similarity to the previously annotated gene ENSG00000249122 are depicted in green.

Transcript ID	Transcript name	Length (nt)	N° of exons	Exons length (nt)
ENST00000508038.2	PHOX2B-AS1-201	973	5	Exon 1: 294<br>Exon 2: 135<br>Exon 3: 159<br>Exon 4: 83<br>Exon 5: 302
ENST00000510602.1	PHOX2B-AS1-202	561	3	Exon 1: 53<br>Exon 2: 117<br>Exon 3: 391
ENST00000819353.1	PHOX2B-AS1-203	921	5	Exon 1: 241<br>Exon 2: 135<br>Exon 3: 159<br>Exon 4: 100<br>Exon 5: 286
ENST00000819354.1	PHOX2B-AS1-204	1070	6	Exon 1: 238<br>Exon 2: 135<br>Exon 3: 159<br>Exon 4: 100<br>Exon 5: 162<br>Exon 6: 276
ENST00000819355.1	PHOX2B-AS1-205	779	5	Exon 1: 235<br>Exon 2: 135<br>Exon 3: 159<br>Exon 4: 100<br>Exon 5: 150
ENST00000819356.1	PHOX2B-AS1-206	1764	5	Exon 1: 238<br>Exon 2: 143<br>Exon 3: 135<br>Exon 4: 159<br>Exon 5: 1089
ENST00000819357.1	PHOX2B-AS1-207	1143	4	Exon 1: 143<br>Exon 2: 135<br>Exon 3: 159<br>Exon 4: 706
ENST00000819358.1	PHOX2B-AS1-208	718	4	Exon 1: 238<br>Exon 2: 159<br>Exon 3: 83<br>Exon 4: 238
ENST00000819359.1	PHOX2B-AS1-209	826	5	Exon 1: 213<br>Exon 2: 135<br>Exon 3: 159<br>Exon 4: 83<br>Exon 5: 236
ENST00000819360.1	PHOX2B-AS1-210	1121	5	Exon 1: 238<br>Exon 2: 143<br>Exon 3: 135<br>Exon 4: 159<br>Exon 5: 446

ENST00000819361.1	PHOX2B-AS1-211	803	4	Exon 1: 238<br>Exon 2: 135<br>Exon 3: 159<br>Exon 4: 271
ENST00000819362.1	PHOX2B-AS1-212	1014	5	Exon 1: 238<br>Exon 2: 143<br>Exon 3: 135<br>Exon 4: 159<br>Exon 5: 339
ENST00000819363.1	PHOX2B-AS1-213	1755	3	Exon 1: 238<br>Exon 2: 135<br>Exon 3: 1382
ENST00000819364.1	PHOX2B-AS1-214	1235	4	Exon 1: 238<br>Exon 2: 135<br>Exon 3: 159<br>Exon 4: 703
ENST00000819365.1	PHOX2B-AS1-215	962	7	Exon 1: 33<br>Exon 2: 117<br>Exon 3: 71<br>Exon 4: 109<br>Exon 5: 135<br>Exon 6: 159<br>Exon 7: 338
ENST00000819366.1	PHOX2B-AS1-216	580	3	Exon 1: 75<br>Exon 2: 133<br>Exon 3: 372
ENST00000819367.1	PHOX2B-AS1-217	564	3	Exon 1: 75<br>Exon 2: 117<br>Exon 3: 372
ENST00000819368.1	PHOX2B-AS1-218	578	3	Exon 1: 63<br>Exon 2: 143<br>Exon 3: 372
ENST00000819369.1	PHOX2B-AS1-219	605	3	Exon 1: 51<br>Exon 2: 191<br>Exon 3: 363
ENST00000819370.1	PHOX2B-AS1-220	747	4	Exon 1: 131<br>Exon 2: 120<br>Exon 3: 133<br>Exon 4: 363
ENST00000819371.1	PHOX2B-AS1-221	580	2	Exon 1: 238<br>Exon 2: 342

Figure S4. Predicted Open Reading Frames and ATGpr Analysis of *PHOX2B-AS1*, related to Figure 1
(A) Predicted amino acid sequences for the three forward reading frames of *PHOX2B-AS1.* (B) Summary table of ATGpr analysis results for *PHOX2B-AS1*. The reliability score reflects the likelihood that a given start codon is in an optimal context for translation initiation, with values closer to 1 indicating higher confidence.

(A)

5'3' Frame 1
F S N A I K A S Y I R A E R R L P P L R K L K V L T C G V R T A L V V P E G A Q A A G S R R E G R A A
G R G P K S G P Y R V I L E A T G L A A G T E V S I G Stop S Q A R G V H P S H T G L V G R G I E G S H
N Q L K E N K T F S C F I T K V L L T L T A T F G R G I L A I D V P G K R V V E E K K L K T W K K D E N
A G Y Stop I P **Met C G Y Stop** V W Y T W T P S S P T H Q **Met S Q R V L L L L D N F K S N Stop** P P Q I
L R S K I I N N N K T C L V N **Met I Y I D Y V G Met F Stop** R E K K G A V T N S K Q H E S K N H A N F A
V C L S Y V L G A K S V V E V H L P E Stop V T K A L Stop L E H F E Y I D L A Stop I S L L **Met A T L T C
Q A I T K Stop** F V C L

5'3' Frame 2
F Q **Met Q S R L P I Y G R K G G F L R Stop** E S Stop R S L P A A Y G L L W W S L R V P R L Q D P G V
R E G Q P D V A P K V V L I G L Y W K P L A W L Q E L K S A Stop A E A R L E V S I P A I Q D S Stop A E
E L R V L T T N Stop K R I K H S L A S Stop P K C C S H L Q Q P L E G E S W L L **Met S L E R E W Stop**
K K R N Stop R H G R K **Met K T L A T K Y L C V V T E C G I L G H R A P P P I R C H K E S S Y S W I I S
N Q T D L P R Y Stop** E A K Stop S T T T K P A L Stop I Stop F T L I **Met W G C S E G R R K E L S P T Q
S N Met R A R I Met L I L P S A S P Met F L V P S L Stop** L R F I Y L N E Stop L K L Y N L N T L N T Stop
I Stop H K Stop A Y Stop W Q L Stop P V K Q Stop P N D L C A C

5'3' Frame 3
F K C N Q G F L Y T G G K A A S S A E K A E G P Y L R R T D C S G G P Stop G C P G C R I P A Stop G
K G S R T W P Q K W S L S G Y T G S H W P G C R N Stop S Q H R L K P G S R C P S Q P Y R T R R Q
R N Stop G F S Q P I E R E Stop N I L L L H N Q S A A H T Y S N L W K G N P G Y Stop C P W K E S G R
R K E I E D **Met E E R Stop** K R W L L N T Y V W L L S V V Y L D T E L P H P S D V T K S P L T L G Stop
F Q I K L T S P D I E K Q N N Q Q Q Q N L P C K Y D L H Stop L C G D V L K G E E R S C H Q L K A T Stop
E Q E S C Stop F C R L P L L C S W C Q V C S Stop G S F T Stop **Met S D Stop** S F I T Stop T L Stop I H R
S S I N K P I N G N F D L S S N N Q **Met I C V P**

(B)

Nº of ATG from 5'end	Reliability	Frame	Identity to Kozak rule A/GXXATGG	Start (bp)	Finish (bp)	ORF Length (aa)	Stop codon found?	Sequence
1	0.15	2	cXXATGc	8	55	16	Yes	MQSRLPIYGRKGGFLR
2	0.04	2	tXXATGt	398	418	7	Yes	MSLEREW
3	0.04	3	GXXATGG	441	452	4	Yes	MEER
4	0.04	2	AXXATGa	452	592	47	Yes	MKTLATKYLCVVTECGILGHRAPPPIRCHKE SSYSWIISNQTDLPRY
5	0.04	1	cXXATGt	478	489	4	Yes	MCGY

Figure S5. Sequences of human *PHOX2B-AS1* splicing variants based on PCR sequencing, related to Figure 2

Exonic regions are indicated by alternating black and blue nucleotide sequences. The extra exon 1a is indicated in lowercase red letters.

PHOX2B-AS1

```
  1 TTTTCAAATGCAATCAAGGCTTCCTATATACGGGCGGAAAGGCGGCTTCCTCCGCTGAGA
 61 AAGCTGAAGGTCCTTACCTGCGGCGTACGGACTGCTCTGGTGGTCCCTGAGGGTGCCCAG
121 GCTGCAGGATCCCGGCGTGAGGGAAGGGCAGCCGGACGTGGCCCCAAAAGTGGTCCTTAT
181 CGGGTTATACTGGAAGCCACTGGCCTGGCTGCAGGAACTGAAGTCAGCATAGGCTGAAGC
241 CAGGCTCGAGGTGTCCATCCCAGCCATACAGGACTCGTAGGCAGAGGAATTGAGGGTTCT
301 CACAACCAATTGAAAGAGAATAAAACATTCTCTTGCTTCATAACCAAAGTGCTGCTCACA
361 CTTACAGCAACCTTTGGAAGGGGAATCCTGGCTATTGATGTCCCTGGAAAGAGAGTGGTA
421 GAAGAAAAGAAATTGAAGACATGGAAGAAAGATGAAAACGCTGGCTACTAAATACCTATG
481 TGTGGTTACTGAGTGTGGTATACTTGGACACCGAGCTCCCCCACCCATCAGATGTCACAA
541 AGAGTCCTCTTACTCTTGGATAATTTCAAATCAAACTGACCTCCCCAGATATTGAGAAGC
601 AAAATAATCAACAACAACAAAACCTGCCTTGTAAATATGATTTACATTGATTATGTGGGG
661 ATGTTCTGAAGGGAGAAGAAAGGAGCTGTCACCAACTCAAAGCAACATGAGAGCAAGAAT
721 CATGCTAATTTTGCCGTCTGCCTCTCCTATGTTCTTGGTGCCAAGTCTGTAGTTGAGGTT
781 CATTTACCTGAATGAGTGACTAAAGCTTTATAACTTGAACACTTTGAATACATAGATCTA
841 GCATAAATAAGCCTATTAATGGCAACTTTGACCTGTCAAGCAATAACCAAATGATTTGTG
901 TGCCTGC
```

PHOX2B-AS1 (1a)

```
   1 TTTTCAAATGCAATCAAGGCTTCCTATATACGGGCGGAAAGGCGGCTTCCTCCGCTGAGA
  61 AAGCTGAAGGTCCTTACCTGCGGCGTACGGACTGCTCTGGTGGTCCCTGAGGGTGCCCAG
 121 GCTGCAGGATCCCGGCGTGAGGGAAGGGCAGCCGGACGTGGCCCCAAAAGTGGTCCTTAT
 181 CGGGTTATACTGGAAGCCACTGGCCTGGCTGCAGGAACTGAAGTCAGCATAGGCTGAAGC
 241 CAGGCTCGAGGTGTCCATCCCAGCCATACAGGACTCGTAGGCAGAGGAATTGAGggagaa
 301 gaaaccttgatgtcatagaaaacctgttttgtaagagtgttacacgctcaatttaacaac
 361 aagcctacccccgaagtgcatgaaatgttataaaggactgggacattggcaagactcag
 421 gcgccctgtaacatagaGGTTCTCACAACCAATTGAAAGAGAATAAAACATTCTCTTGCT
 481 TCATAACCAAAGTGCTGCTCACACTTACAGCAACCTTTGGAAGGGGAATCCTGGCTATTG
 541 ATGTCCCTGGAAAGAGAGTGGTAGAAGAAAAGAAATTGAAGACATGGAAGAAAGATGAAA
 601 ACGCTGGCTACTAAATACCTATGTGTGGTTACTGAGTGTGGTATACTTGGACACCGAGCT
 661 CCCCCACCCATCAGATGTCACAAAGAGTCCTCTTACTCTTGGATAATTTCAAATCAAACT
 721 GACCTCCCCAGATATTGAGAAGCAAAATAATCAACAACAACAAAACCTGCCTTGTAAATA
 781 TGATTTACATTGATTATGTGGGGATGTTCTGAAGGGAGAAGAAAGGAGCTGTCACCAACT
 841 CAAAGCAACATGAGAGCAAGAATCATGCTAATTTTGCCGTCTGCCTCTCCTATGTTCTTG
 901 GTGCCAAGTCTGTAGTTGAGGTTCATTTACCTGAATGAGTGACTAAAGCTTTATAACTTG
 961 AACACTTTGAATACATAGATCTAGCATAAATAAGCCTATTAATGGCAACTTTGACCTGTC
1021 AAGCAATAACCAAATGATTTGTGTGCCTGC
```

PHOX2B-AS1 (2a)

```
  1 TTTTCAAATGCAATCAAGGCTTCCTATATACGGGCGGAAAGGCGGCTTCCTCCGCTGAGA
 61 AAGCTGAAGGTCCTTACCTGCGGCGTACGGACTGCTCTGGTGGTCCCTGAGGGTGCCCAG
121 GCTGCAGGATCCCGGCGTGAGGGAAGGGCAGCCGGACGTGGCCCCAAAAGTGGTCCTTAT
181 CGGGTTATACTGGAAGCCACTGGCCTGGCTGCAGGAACTGAAGTCAGCATAGGCTGAAGC
241 CAGGCTCGAGGTGTCCATCCCAGCCATACAGGACTCGTAGGCAGAGGAATTGAGCAACCT
301 TTGGAAGGGGAATCCTGGCTATTGATGTCCCTGGAAAGAGAGTGGTAGAAGAAAAGAAAT
361 TGAAGACATGGAAGAAAGATGAAAACGCTGGCTACTAAATACCTATGTGTGGTTACTGAG
421 TGTGGTATACTTGGACACCGAGCTCCCCCACCCATCAGATGTCACAAAGAGTCCTCTTAC
481 TCTTGGATAATTTCAAATCAAACTGACCTCCCCAGATATTGAGAAGCAAAATAATCAACA
541 ACAACAAAACCTGCCTTGTAAATATGATTTACATTGATTATGTGGGGATGTTCTGAAGGG
601 AGAAGAAAGGAGCTGTCACCAACTCAAAGCAACATGAGAGCAAGAATCATGCTAATTTTG
661 CCGTCTGCCTCTCCTATGTTCTTGGTGCCAAGTCTGTAGTTGAGGTTCATTTACCTGAAT
721 GAGTGACTAAAGCTTTATAACTTGAACACTTTGAATACATAGATCTAGCATAAATAAGCC
781 TATTAATGGCAACTTTGACCTGTCAAGCAATAACCAAATGATTTGTGTGCCTGC
```

PHOX2B-AS1 (1a/2a)

```
  1 TTTTCAAATGCAATCAAGGCTTCCTATATACGGGCGGAAAGGCGGCTTCCTCCGCTGAGA
 61 AAGCTGAAGGTCCTTACCTGCGGCGTACGGACTGCTCTGGTGGTCCCTGAGGGTGCCCAG
121 GCTGCAGGATCCCGGCGTGAGGGAAGGGCAGCCGGACGTGGCCCCAAAAGTGGTCCTTAT
181 CGGGTTATACTGGAAGCCACTGGCCTGGCTGCAGGAACTGAAGTCAGCATAGGCTGAAGC
241 CAGGCTCGAGGTGTCCATCCCAGCCATACAGGACTCGTAGGCAGAGGAATTGAGggagaa
301 gaaaccttgatgtcatagaaaacctgttttgtaagagtgttacacgctcaatttaacaac
361 aagcctacccccgaagtgcatgaaatgttataaaggactgggacattggcaagactcag
421 gcgccctgtaacatagaCAACCTTTGGAAGGGGAATCCTGGCTATTGATGTCCCTGGAAA
481 GAGAGTGGTAGAAGAAAAGAAATTGAAGACATGGAAGAAAGATGAAAACGCTGGCTACTA
541 AATACCTATGTGTGGTTACTGAGTGTGGTATACTTGGACACCGAGCTCCCCCACCCATCA
601 GATGTCACAAAGAGTCCTCTTACTCTTGGATAATTTCAAATCAAACTGACCTCCCCAGAT
661 ATTGAGAAGCAAAATAATCAACAACAACAAAACCTGCCTTGTAAATATGATTTACATTGA
721 TTATGTGGGGATGTTCTGAAGGGAGAAGAAAGGAGCTGTCACCAACTCAAAGCAACATGA
781 GAGCAAGAATCATGCTAATTTTGCCGTCTGCCTCTCCTATGTTCTTGGTGCCAAGTCTGT
841 AGTTGAGGTTCATTTACCTGAATGAGTGACTAAAGCTTTATAACTTGAACACTTTGAATA
901 CATAGATCTAGCATAAATAAGCCTATTAATGGCAACTTTGACCTGTCAAGCAATAACCAA
961 ATGATTTGTGTGCCTGC
```

```
CLUSTAL O(1.2.4) multiple sequence alignment

PHOX2B-AS1             TTTTCAAATGCAATCAAGGCTTCCTATATACGGGCGGAAAGGCGGCTTCCTCCGCTGAGA   60
PHOX2B-AS1_1a          TTTTCAAATGCAATCAAGGCTTCCTATATACGGGCGGAAAGGCGGCTTCCTCCGCTGAGA   60
PHOX2B-AS1_2a          TTTTCAAATGCAATCAAGGCTTCCTATATACGGGCGGAAAGGCGGCTTCCTCCGCTGAGA   60
PHOX2B-AS1_1a/2a       TTTTCAAATGCAATCAAGGCTTCCTATATACGGGCGGAAAGGCGGCTTCCTCCGCTGAGA   60
                       ************************************************************

PHOX2B-AS1             AAGCTGAAGGTCCTTACCTGCGGCGTACGGACTGCTCTGGTGGTCCCTGAGGGTGCCCAG   120
PHOX2B-AS1_1a          AAGCTGAAGGTCCTTACCTGCGGCGTACGGACTGCTCTGGTGGTCCCTGAGGGTGCCCAG   120
PHOX2B-AS1_2a          AAGCTGAAGGTCCTTACCTGCGGCGTACGGACTGCTCTGGTGGTCCCTGAGGGTGCCCAG   120
PHOX2B-AS1_1a/2a       AAGCTGAAGGTCCTTACCTGCGGCGTACGGACTGCTCTGGTGGTCCCTGAGGGTGCCCAG   120
                       ************************************************************

PHOX2B-AS1             GCTGCAGGATCCCGGCGTGAGGGAAGGGCAGCCGGACGTGGCCCCAAAAGTGGTCCTTAT   180
PHOX2B-AS1_1a          GCTGCAGGATCCCGGCGTGAGGGAAGGGCAGCCGGACGTGGCCCCAAAAGTGGTCCTTAT   180
PHOX2B-AS1_2a          GCTGCAGGATCCCGGCGTGAGGGAAGGGCAGCCGGACGTGGCCCCAAAAGTGGTCCTTAT   180
PHOX2B-AS1_1a/2a       GCTGCAGGATCCCGGCGTGAGGGAAGGGCAGCCGGACGTGGCCCCAAAAGTGGTCCTTAT   180
                       ************************************************************

PHOX2B-AS1             CGGGTTATACTGGAAGCCACTGGCCTGGCTGCAGGAACTGAAGTCAGCATAGGCTGAAGC   240
PHOX2B-AS1_1a          CGGGTTATACTGGAAGCCACTGGCCTGGCTGCAGGAACTGAAGTCAGCATAGGCTGAAGC   240
PHOX2B-AS1_2a          CGGGTTATACTGGAAGCCACTGGCCTGGCTGCAGGAACTGAAGTCAGCATAGGCTGAAGC   240
PHOX2B-AS1_1a/2a       CGGGTTATACTGGAAGCCACTGGCCTGGCTGCAGGAACTGAAGTCAGCATAGGCTGAAGC   240
                       ************************************************************

PHOX2B-AS1             CAGGCTCGAGGTGTCCATCCCAGCCATACAGGACTCGTAGGCAGAGGAATTGAG------   295
PHOX2B-AS1_1a          CAGGCTCGAGGTGTCCATCCCAGCCATACAGGACTCGTAGGCAGAGGAATTGAGggagaa   300
PHOX2B-AS1_2a          CAGGCTCGAGGTGTCCATCCCAGCCATACAGGACTCGTAGGCAGAGGAATTGAG------   294
PHOX2B-AS1_1a/2a       CAGGCTCGAGGTGTCCATCCCAGCCATACAGGACTCGTAGGCAGAGGAATTGAGggagaa   300
                       ******************************************************

PHOX2B-AS1             ------------------------------------------------------------   295
PHOX2B-AS1_1a          gaaaccttgatgtcatagaaaacctgttttgtaagagtgttacacgctcaatttaacaac   360
PHOX2B-AS1_2a          ------------------------------------------------------------   294
PHOX2B-AS1_1a/2a       gaaaccttgatgtcatagaaaacctgttttgtaagagtgttacacgctcaatttaacaac   360


PHOX2B-AS1             ------------------------------------------------------------   295
PHOX2B-AS1_1a          aagcctacccccgaagtgcatgaaatgttataaaggactgggacattggcaagactcag   420
PHOX2B-AS1_2a          ------------------------------------------------------------   294
PHOX2B-AS1_1a/2a       aagcctacccccgaagtgcatgaaatgttataaaggactgggacattggcaagactcag   420


PHOX2B-AS1             -----------------GGTTCTCACAACCAATTGAAAGAGAATAAAACATTCTCTTGCT   337
PHOX2B-AS1_1a          gcgccctgtaacatagaGGTTCTCACAACCAATTGAAAGAGAATAAAACATTCTCTTGCT   480
```

```
PHOX2B-AS1_2a         ------------------------------------------------------------   294
PHOX2B-AS1_1a/2a      gcgccctgtaacataga-------------------------------------------   437

PHOX2B-AS1            TCATAACCAAAGTGCTGCTCACACTTACAGCAACCTTTGGAAGGGGAATCCTGGCTATTG   397
PHOX2B-AS1_1a         TCATAACCAAAGTGCTGCTCACACTTACAGCAACCTTTGGAAGGGGAATCCTGGCTATTG   540
PHOX2B-AS1_2a         ------------------------------CAACCTTTGGAAGGGGAATCCTGGCTATTG   324
PHOX2B-AS1_1a/2a      ------------------------------CAACCTTTGGAAGGGGAATCCTGGCTATTG   467
                                                    ******************************

PHOX2B-AS1            ATGTCCCTGGAAAGAGAGTGGTAGAAGAAAAGAAATTGAAGACATGGAAGAAAGATGAAA   457
PHOX2B-AS1_1a         ATGTCCCTGGAAAGAGAGTGGTAGAAGAAAAGAAATTGAAGACATGGAAGAAAGATGAAA   600
PHOX2B-AS1_2a         ATGTCCCTGGAAAGAGAGTGGTAGAAGAAAAGAAATTGAAGACATGGAAGAAAGATGAAA   384
PHOX2B-AS1_1a/2a      ATGTCCCTGGAAAGAGAGTGGTAGAAGAAAAGAAATTGAAGACATGGAAGAAAGATGAAA   527
                      ************************************************************

PHOX2B-AS1            ACGCTGGCTACTAAATACCTATGTGTGGTTACTGAGTGTGGTATACTTGGACACCGAGCT   517
PHOX2B-AS1_1a         ACGCTGGCTACTAAATACCTATGTGTGGTTACTGAGTGTGGTATACTTGGACACCGAGCT   660
PHOX2B-AS1_2a         ACGCTGGCTACTAAATACCTATGTGTGGTTACTGAGTGTGGTATACTTGGACACCGAGCT   444
PHOX2B-AS1_1a/2a      ACGCTGGCTACTAAATACCTATGTGTGGTTACTGAGTGTGGTATACTTGGACACCGAGCT   587
                      ************************************************************

PHOX2B-AS1            CCCCCACCCATCAGATGTCACAAAGAGTCCTCTTACTCTTGGATAATTTCAAATCAAACT   577
PHOX2B-AS1_1a         CCCCCACCCATCAGATGTCACAAAGAGTCCTCTTACTCTTGGATAATTTCAAATCAAACT   720
PHOX2B-AS1_2a         CCCCCACCCATCAGATGTCACAAAGAGTCCTCTTACTCTTGGATAATTTCAAATCAAACT   504
PHOX2B-AS1_1a/2a      CCCCCACCCATCAGATGTCACAAAGAGTCCTCTTACTCTTGGATAATTTCAAATCAAACT   647
                      ************************************************************

PHOX2B-AS1            GACCTCCCCAGATATTGAGAAGCAAAATAATCAACAACAACAAAACCTGCCTTGTAAATA   637
PHOX2B-AS1_1a         GACCTCCCCAGATATTGAGAAGCAAAATAATCAACAACAACAAAACCTGCCTTGTAAATA   780
PHOX2B-AS1_2a         GACCTCCCCAGATATTGAGAAGCAAAATAATCAACAACAACAAAACCTGCCTTGTAAATA   564
PHOX2B-AS1_1a/2a      GACCTCCCCAGATATTGAGAAGCAAAATAATCAACAACAACAAAACCTGCCTTGTAAATA   707
                      ************************************************************

PHOX2B-AS1            TGATTTACATTGATTATGTGGGGATGTTCTGAAGGGAGAAGAAAGGAGCTGTCACCAACT   697
PHOX2B-AS1_1a         TGATTTACATTGATTATGTGGGGATGTTCTGAAGGGAGAAGAAAGGAGCTGTCACCAACT   840
PHOX2B-AS1_2a         TGATTTACATTGATTATGTGGGGATGTTCTGAAGGGAGAAGAAAGGAGCTGTCACCAACT   624
PHOX2B-AS1_1a/2a      TGATTTACATTGATTATGTGGGGATGTTCTGAAGGGAGAAGAAAGGAGCTGTCACCAACT   767
                      ************************************************************

PHOX2B-AS1            CAAAGCAACATGAGAGCAAGAATCATGCTAATTTTGCCGTCTGCCTCTCCTATGTTCTTG   757
PHOX2B-AS1_1a         CAAAGCAACATGAGAGCAAGAATCATGCTAATTTTGCCGTCTGCCTCTCCTATGTTCTTG   900
PHOX2B-AS1_2a         CAAAGCAACATGAGAGCAAGAATCATGCTAATTTTGCCGTCTGCCTCTCCTATGTTCTTG   684
PHOX2B-AS1_1a/2a      CAAAGCAACATGAGAGCAAGAATCATGCTAATTTTGCCGTCTGCCTCTCCTATGTTCTTG   827
                      ************************************************************

PHOX2B-AS1            GTGCCAAGTCTGTAGTTGAGGTTCATTTACCTGAATGAGTGACTAAAGCTTTATAACTTG   817
PHOX2B-AS1_1a         GTGCCAAGTCTGTAGTTGAGGTTCATTTACCTGAATGAGTGACTAAAGCTTTATAACTTG   960
PHOX2B-AS1_2a         GTGCCAAGTCTGTAGTTGAGGTTCATTTACCTGAATGAGTGACTAAAGCTTTATAACTTG   744
PHOX2B-AS1_1a/2a      GTGCCAAGTCTGTAGTTGAGGTTCATTTACCTGAATGAGTGACTAAAGCTTTATAACTTG   887
                      ************************************************************

PHOX2B-AS1            AACACTTTGAATACATAGATCTAGCATAAATAAGCCTATTAATGGCAACTTTGACCTGTC   877
PHOX2B-AS1_1a         AACACTTTGAATACATAGATCTAGCATAAATAAGCCTATTAATGGCAACTTTGACCTGTC   1020
PHOX2B-AS1_2a         AACACTTTGAATACATAGATCTAGCATAAATAAGCCTATTAATGGCAACTTTGACCTGTC   804
PHOX2B-AS1_1a/2a      AACACTTTGAATACATAGATCTAGCATAAATAAGCCTATTAATGGCAACTTTGACCTGTC   947
                      ************************************************************

PHOX2B-AS1            AAGCAATAACCAAATGATTTGTGTGCCTGC    907
PHOX2B-AS1_1a         AAGCAATAACCAAATGATTTGTGTGCCTGC    1050
PHOX2B-AS1_2a         AAGCAATAACCAAATGATTTGTGTGCCTGC    834
PHOX2B-AS1_1a/2a      AAGCAATAACCAAATGATTTGTGTGCCTGC    977
                      ******************************
```

Figure S6. Genomic location of the *Phox2b* and *Gm33167* transcripts as annotated in the UCSC Genome Browser, related to Figure 3

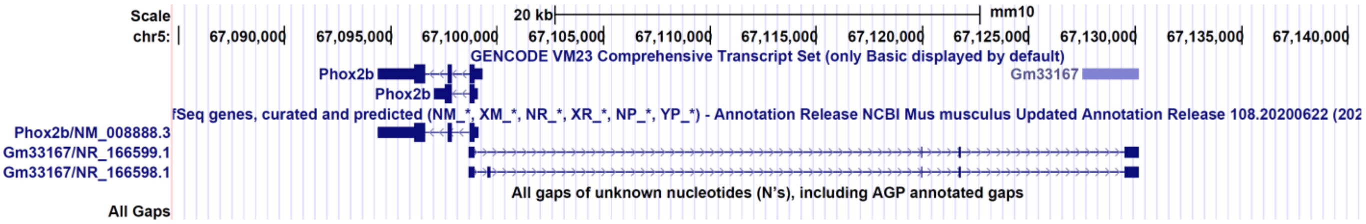

Gm33167 splicing variants sequences. Exonic regions are indicated by alternating colored nucleotide sequences.

```
>Mus musculus predicted gene, 33167 (Gm33167), transcript variant 1,
            long non-coding RNA. NR_166598.1

      1 gagagagcta gcggtcctta cctgcggcgt acggactgct ctggtggtcc ctgagggtgc
     61 ccaggctgca ggatcccggc gtgagggacg ggcaaccgga cgtggcccca aaagtggtcc
    121 ttatcgggtt atactggaag ccactggcct ggctgcagga actgaagtct gcatatgctg
    181 aagccaagct ggaggtatcc atcccggcca tacaggactc gtaggcagag gaattgaggg
    241 agaagaaacc ttgatgtcat agaaaacctg ttttgtaaga gtgttacacg ctcaatttaa
    301 caacaagcct tcccccgaag tgcatgaaat gctataaagg actgggacat tggcaagact
    361 caggcgccct gtaacatagc cacctcccaa gactacatgt gactggagca gatttacaaa
    421 cagcagggag agagctgcag gtggtgctgg aatctcagac atagctcatt gccaaggacc
    481 tcaagaagtc attgaatcca tccctctgcc tccaggcctg cttgctcctc caatcaattc
    541 ttgataaaga agagagaata aggcgggact tccaccagcc agaggaagga gaaagattga
    601 agtttgagcc acagaggaag gtgtctggaa agggatcatg gaaacccacc tgaagcccaa
    661 aaagcgagat gaataataaa attcaaatcc aagtatcatg agggaagttg gtcgagaggt
    721 agccaattag cttagggaaa ttattataag tagtttgact gtccagatac cgaggtgaag
    781 gcttaaaaca aaacttttgt ctttctttgt tattatttgg aactagctag gataaagaca
    841 tacaaccact gtattaattt actacttaaa tgtatattaa aagttatatt tacaaaaaat
    901 aataaattaa gaatataata aagcaaggca gatctcagat gagtaaaaca gaatgctact
    961 actgtattcc attgcggtaa agggaatcat gggagtgtga gcatcgccac gggaggctgg
   1021 caatggtggt ctcttgttag aactgtgagc gacattttct tccttgtact tttctgagat
   1081 ttccaaggat tctctgtaaa catgttatgt tttataactg ggaaaaacat gcattaatct
   1141 tcccctctct tattcagttt caataaaaag atactacttt ggtttgttca ta

>Mus musculus predicted gene, 33167 (Gm33167), transcript variant 2,
            long non-coding RNA. NR_166599.1

      1 gagagagcta gcggtcctta cctgcggcgt acggactgct ctggtggtcc ctgagggtgc
     61 ccaggctgca ggatcccggc gtgagggacg ggcaaccgga cgtggcccca aaagtggtcc
    121 ttatcgggtt atactggaag ccactggcct ggctgcagga actgaagtct gcatatgctg
    181 aagccaagct ggaggtatcc atcccggcca tacaggactc gtaggcagag gaattgagca
    241 cctcccaaga ctacatgtga ctggagcaga tttacaaaca gcagggagag agctgcaggt
    301 ggtgctggaa tctcagacat agctcattgc caaggacctc aagaagtcat tgaatccatc
    361 cctctgcctc caggcctgct tgctcctcca atcaattctt gataaagaag agagaataag
    421 gcgggacttc caccagccag aggaaggaga aagattgaag tttgagccac agaggaaggt
    481 gtctggaaag ggatcatgga aacccacctg aagcccaaaa agcgagatga ataataaaat
    541 tcaaatccaa gtatcatgag ggaagttggt cgagaggtag ccaattagct tagggaaatt
    601 attataagta gtttgactgt ccagataccg aggtgaaggc ttaaaacaaa acttttgtct
    661 ttctttgtta ttatttggaa ctagctagga taaagacata caaccactgt attaatttac
    721 tacttaaatg tatattaaaa gttatattta caaaaaataa taaattaaga atataataaa
    781 gcaaggcaga tctcagatga gtaaaacaga atgctactac tgtattccat tgcggtaaag
    841 ggaatcatgg gagtgtgagc atcgccacgg gaggctggca atggtggtct cttgttagaa
    901 ctgtgagcga cattttcttc cttgtacttt tctgagattt ccaaggattc tctgtaaaca
    961 tgttatgttt tataactggg aaaaacatgc attaatcttc ccctctctta ttcagtttca
   1021 ataaaaagat actactttgg tttgttcata
```

Figure S7. Sequence of mouse *Phox2b-As* identified by 5' and 3' RACE, related to Figure 3

The yellow highlighted region indicates the mAs 3′ RACE outer primer. The underlined uppercase sequence denotes the region overlapping with the mouse *Phox2b* transcript. Red text represents exon 1, while blue text indicates exon 2.

```
TGAGAGAGCTAGCGGTCCTTACCTGCGGCGTACGGACTGCTCTGGTGGTCCCTGAGGGTGCCCAGGCTGCAGG
ATCCCGGCGTGAGGGACGGGCAACCGGACGTGGCCCCAAAAGTGGTCCTTATCGGGTTATACTGGAAGCCACT
GGCCTGGCTGCAGGAACTGAAGTCTGCATATGCTGAAGCCAAGCTGGAGGTATCCATCCCGGCCATACAGGAC
TCGTAGGCAGAGGAATTGAGGGAGAAGAAACCTTGATGTCATAGAAAACCTGTTTTGTAAGAGTGTTACACGC
TCAATTTAACAACAAGCCTACCCCCTGAAGTGCATGAAATGCTATAAAGGACTGGGACATTGGCAAGACTCAG
GCGCCCTGTAACATAGCGTAAGTGGGCCAAAGGGTTTTATGTGAACGATAAGGATAAGCGGATTTCTTTAAAA
TACACCTAGTCCAAGGGGTTGGAAAAAATAGCATCTTATCAATTGTGTTTCTCCTCGAAGCTTAATGTGCTTT
CCAAAGACGTGGGCAGACAGCCCCTTTACAAGAGTAAAATAAACAACCGAGTAAAGCTCCCAAAAAAAAAAA
```

Figure S8. Alignment of *Phox2b-As* with *Gm33167* transcript variant 1, related to Figure 3

The yellow highlighted region corresponds to the first exon of *Phox2b-As* aligned with *Gm33167* transcript variant 1. The green highlighted region indicates the second exon of *Phox2b-As* aligned with *Gm33167* transcript variant 1

```
CLUSTAL O(1.2.4) multiple sequence alignment
Phox2b-As      TGAGAGAGCTAGCGGTCCTTACCTGCGGCGTACGGACTGCTCTGGTGGTCCCTGAGGGTG    60
Gm33167        -gagagagctagcggtccttacctgcggcgtacggactgctctggtggtccctgagggtg    59
                ***********************************************************

Phox2b-As      CCCAGGCTGCAGGATCCCGGCGTGAGGGACGGGCAACCGGACGTGGCCCCAAAAGTGGTC    120
Gm33167        cccaggctgcaggatcccggcgtgagggacgggcaaccggacgtggccccaaaagtggtc    119
               ************************************************************

Phox2b-As      CTTATCGGGTTATACTGGAAGCCACTGGCCTGGCTGCAGGAACTGAAGTCTGCATATGCT    180
Gm33167        cttatcgggttatactggaagccactggcctggctgcaggaactgaagtctgcatatgct    179
               ************************************************************

Phox2b-As      GAAGCCAAGCTGGAGGTATCCATCCCGGCCATACAGGACTCGTAGGCAGAGGAATTGAGG    240
Gm33167        gaagccaagctggaggtatccatcccggccatacaggactcgtaggcagaggaattgagg    239
               ************************************************************

Phox2b-As      GAGAAGAAACCTTGATGTCATAGAAAACCTGTTTTGTAAGAGTGTTACACGCTCAATTTA    300
Gm33167        gagaagaaaccttgatgtcatagaaaacctgttttgtaagagtgttacacgctcaattta    299
               ************************************************************

Phox2b-As      ACAACAAGCCTACCCCCTGAAGTGCATGAAATGCTATAAAGGACTGGGACATTGGCAAGA    360
Gm33167        acaacaagccttccc-ccgaagtgcatgaaatgctataaaggactgggacattggcaaga    358
               *********** *** * ******************************************

Phox2b-As      CTCAGGCGCCCTGTAACATAGCGTAAGTGGGCCAAAGGGTTTTATGTGAACGATAAGGAT    420
Gm33167        ctcaggcgccctgtaacatagccacctcccaagactacatgtgactggagcagatttaca    418
               **********************           *     * * *   ** *

Phox2b-As      AAGCGGATTTCTTTAAAATACACCTAGTCCAAGGGGTTGGAAAAAATAGCATCTTATCA-    479
Gm33167        aac-agcagggagagagctgcaggtggtgctggaatct--cagacatagctcattgccaa    475
               **   *         *  * **  * ** *  *    *   * * *****   **  **

Phox2b-As      -----------AT----TGTGT------TTC-TCCTCGAAGCTTAATGTGCTTTCCAA--    515
Gm33167        ggacctcaagaagtcattgaatccatccctctgcctccaggcctgcttgctcctccaatc    535
                          *     **  *       **  **** * ** *  *      *****

Phox2b-As      ------------AGACGTGGGCAGACAG---CCCCTTTACAAGAGTAAAATAAACAACCG    560
Gm33167        aattcttgataaagaagagagaataaggcgggacttccaccagccagaggaaggagaaag    595
                           *** * * * * *  *     * *  ** **    *   *    *  *

Phox2b-As      AGTAAAGCTCCCAAAAAAAAAAA-------------------------------------    583
Gm33167        attgaagtttgagccacagaggaaggtgtctggaaagggatcatggaaacccacctgaag    655
               * * *** *      * * *  *
```

```
Phox2b-As        ------------------------------------------------------------   583
Gm33167          cccaaaaagcgagatgaataataaaattcaaatccaagtatcatgagggaagttggtcga   715


Phox2b-As        ------------------------------------------------------------   583
Gm33167          gaggtagccaattagcttagggaaattattataagtagtttgactgtccagataccgagg   775


Phox2b-As        ------------------------------------------------------------   583
Gm33167          tgaaggcttaaaacaaaacttttgtctttctttgttattatttggaactagctaggataa   835


Phox2b-As        ------------------------------------------------------------   583
Gm33167          agacatacaaccactgtattaatttactacttaaatgtatattaaaagttatatttacaa   895


Phox2b-As        ------------------------------------------------------------   583
Gm33167          aaaataataaattaagaatataataaagcaaggcagatctcagatgagtaaaacagaatg   955


Phox2b-As        ------------------------------------------------------------   583
Gm33167          ctactactgtattccattgcggtaaagggaatcatgggagtgtgagcatcgccacgggag   1015


Phox2b-As        ------------------------------------------------------------   583
Gm33167          gctggcaatggtggtctcttgttagaactgtgagcgacattttcttccttgtacttttct   1075


Phox2b-As        ------------------------------------------------------------   583
Gm33167          gagatttccaaggattctctgtaaacatgttatgttttataactgggaaaaacatgcatt   1135


Phox2b-As        ---------------------------------------------------------      583
Gm33167          aatcttcccctctcttattcagtttcaataaaaagatactactttggtttgttcata      1192
```

Figure S9. Alignment of mouse *Phox2b-As* with human *PHOX2B-AS1 (1a)*, related to Figure 3

The yellow highlighted region represents the sequence overlapping with the mouse *Phox2b* transcript. The green highlighted region corresponds to *Phox2b-As* exon 2 and *PHOX2B-AS1* exon 1a (in lowercase).

```
CLUSTAL O(1.2.4) multiple sequence alignment

Phox2b-As        -------------------------------------------------------TGAGA   5
PHOX2B-AS1       TTTTCAAATGCAATCAAGGCTTCCTATATACGGGCGGAAAGGCGGCTTCCTCCGCTGAGA   60
                                                                        *****

Phox2b-As        GAGCTAGCGGTCCTTACCTGCGGCGTACGGACTGCTCTGGTGGTCCCTGAGGGTGCCCAG   65
PHOX2B-AS1       AAGCTGAAGGTCCTTACCTGCGGCGTACGGACTGCTCTGGTGGTCCCTGAGGGTGCCCAG   120
                  ****   ****************************************************

Phox2b-As        GCTGCAGGATCCCGGCGTGAGGGACGGCAACCGGACGTGGCCCCAAAAGTGGTCCTTAT   125
PHOX2B-AS1       GCTGCAGGATCCCGGCGTGAGGGAAGGCAGCCGGACGTGGCCCCAAAAGTGGTCCTTAT   180
                 ************************ ***** *****************************

Phox2b-As        CGGGTTATACTGGAAGCCACTGGCCTGGCTGCAGGAACTGAAGTCTGCATATGCTGAAGC   185
PHOX2B-AS1       CGGGTTATACTGGAAGCCACTGGCCTGGCTGCAGGAACTGAAGTCAGCATAGGCTGAAGC   240
                 ********************************************* ***** ********

Phox2b-As        CAAGCTGGAGGTATCCATCCCGGCCATACAGGACTCGTAGGCAGAGGAATTGAGGGAGAA   245
PHOX2B-AS1       CAGGCTCGAGGTGTCCATCCCAGCCATACAGGACTCGTAGGCAGAGGAATTGAGggagaa   300
                 ** *** ***** ******** **************************************

Phox2b-As        GAAACCTTGATGTCATAGAAAACCTGTTTTGTAAGAGTGTTACACGCTCAATTTAACAAC   305
PHOX2B-AS1       gaaaccttgatgtcatagaaaacctgttttgtaagagtgttacacgctcaatttaacaac   360
                 ************************************************************

Phox2b-As        AAGCCTACCCCCTGAAGTGCATGAAATGCTATAAAGGACTGGGACATTGGCAAGACTCAG   365
PHOX2B-AS1       aagcctacccccgaagtgcatgaaatgttataaaggactgggacattggcaagactcag   420
                 ************ *************** *******************************
```

```
Phox2b-As       GCGCCCTGTAACATAGCGTAAGTGGGCCAAAGGGTTTTATGTGAACGATAAGGATAAGCG   425
PHOX2B-AS1      gcgccctgtaacatagaGGTTCTCACAACCAATTGAAAGAGAATAAAAC----ATTCTCT   476
                **************** *    *       *         *   *  *     **   *

Phox2b-As       GATTTCTTTAAAATACACCTAGTCCAAGGGGTTGGAAAAAAT------AGCATCTTATCA   479
PHOX2B-AS1      TGCTTCATAACCAAAGTGCTGCTCACACTTACAGCAACCTTTGGAAGGGGAATCCTGGCT   536
                   *** * *  * *   **  **  *      * **    *       * *** *  *

Phox2b-As       ATTGTGTTTCTCCTCGAAGCTTAATGTGCTTTCCAAAGACGTGGGCAGACAGCCCCTTTA   539
PHOX2B-AS1      AT--TGATGTCCCTGGAAAGAGAGTGGTAGAAGAAAAGAAATTG-AAGACATGGAAGAAA   593
                **  ** *   *** ***    * **        *****  * *  *****        *

Phox2b-As       CAAG-------AGTAAAATAAACAACCGAGTAAAG-------------------------   567
PHOX2B-AS1      GATGAAAACGCTGGCTACTAAATACCTATGTGTGGTTACTGAGTGTGGTATACTTGGACA   653
                 * *        *   * **** * *   **   *

Phox2b-As       -----CTCCCAAAAAAAAAAA---------------------------------------   583
PHOX2B-AS1      CCGAGCTCCCCCACCCATCAGATGTCACAAAGAGTCCTCTTACTCTTGGATAATTTCAAA   713
                     *****  *   *  *

Phox2b-As       ------------------------------------------------------------   583
PHOX2B-AS1      TCAAACTGACCTCCCCAGATATTGAGAAGCAAAATAATCAACAACAACAAAACCTGCCTT   773

Phox2b-As       ------------------------------------------------------------   583
PHOX2B-AS1      GTAAATATGATTTACATTGATTATGTGGGGATGTTCTGAAGGGAGAAGAAAGGAGCTGTC   833

Phox2b-As       ------------------------------------------------------------   583
PHOX2B-AS1      ACCAACTCAAAGCAACATGAGAGCAAGAATCATGCTAATTTTGCCGTCTGCCTCTCCTAT   893

Phox2b-As       ------------------------------------------------------------   583
PHOX2B-AS1      GTTCTTGGTGCCAAGTCTGTAGTTGAGGTTCATTTACCTGAATGAGTGACTAAAGCTTTA   953

Phox2b-As       ------------------------------------------------------------   583
PHOX2B-AS1      TAACTTGAACACTTTGAATACATAGATCTAGCATAAATAAGCCTATTAATGGCAACTTTG   1013

Phox2b-As       -------------------------------------      583
PHOX2B-AS1      ACCTGTCAAGCAATAACCAAATGATTTGTGTGCCTGC      1050
```

Table S1. List and sequences of primers and gapmeRs used for experiments

(F: Forward, R: Reverse)

Primer name	Sequence (5'3')	Application
AS1 5' RACE OUTER	CTCAATTCCTCTGCCTACGAGTCCT	5' RACE
AS1 5' RACE INNER	TTCCTGCAGCCAGGCCAGTGG	5' RACE
AS1 3' RACE OUTER	TACTTGGACACCGAGCTCCC	3' RACE
AS1 3' RACE INNER_1	ATCAACAACAACAAAACCTGCCTT	3' RACE
AS1 3' RACE INNER_2	TGTGGGGATGTTCTGAAGGGAGAA	3' RACE
AS1- F1	CCTCCGCTGAGAAAGCTGAA	PCR
AS1- R1	GCCAGGATTCCCCTTCCAAA	PCR
PHOX2B-F	CGGAATTCCAATGTATAAAATGGAATATTC	PCR
PHOX2B-R	ACTCGCGCCTCTGTGAGGTCG	PCR
AS1- F1_EcoRI	TATGAATTCCCTCCGCTGAGAAAGCTGAA	Cloning
AS1- F2_EcoRI	TATGAATTCTTTTCAAATGCAATCAAGGCTT	Cloning
AS1- R2_EcoRI	TATGAATTCGCAGGCACACAAATCATTTG	Cloning
AS1 RT-ex4	TTCAGAACATCCCCACATAATCAATGT	SS-RT-PCR
AS1 RT-ex5	TTGCTTGACAGGTCAAAGTTGC	SS-RT-PCR
AS1-F qPCR	ACCTTTGGAAGGGGAATCCTGGC	qPCR
AS1-R qPCR	AGCCAGCGTTTTCATCTTTCTTCCA	qPCR
PHOX2B-F qPCR	GTAGGCCCAAGGCTATTGTCGTCGCT	qPCR
PHOX2B-R qPCR	GTAGGAGTGGGGTTGAAATGAGGGCG	qPCR
GAPDH-F qPCR	TCGGAGTCAACGGATTTGG	qPCR
GAPDH-R qPCR	TGGCAACAATATCCACTTTACCA	qPCR
LNA GapmeR control	AACACGTCTATACGC	Silencing (Qiagen, Cat#339515)
PHOX2B-AS1 LNA GapmeR-1	AGGCACACAAATCATT	Silencing (Qiagen, Cat#339511 LG00215307-DDA)
PHOX2B-AS1 LNA GapmeR-2	AGGAAGCCTTGATTGC	Silencing (Qiagen, Cat#339511 LG00215308-DDA)
PHOX2B-AS1 LNA GapmeR-3	CCTTGATTGCATTTGA	Silencing (Qiagen, Cat#339511 LG00215309-DDA)
AS1 promoter +866-F_XhoI	ATACTCGAGTCAGAAAGTTGACCCGGCTC	PCR (Cloning)
AS1 promoter +366-R_BglII	ATAAGATCTTTCAGCTTTCTCAGCGGAGG	PCR (Cloning)
AS prom +2933-F_XhoI	ATACTCGAGCGCAGGATTCCAGATCAG	PCR (Cloning)
AS prom +1440-R	GGCGGCTGGTGAAATTCGAA	PCR (Cloning)
AS prom +1475-F	CCAGTATTTCTGATCGGCCATGG	PCR (Cloning)
mAs 5' RACE OUTER	TGGCTTCAGCATATGCAGACTTCAGT	5' RACE
mAs 5' RACE INNER	CCACTTTTGGGGCCACGTCC	5' RACE
mAs 3' RACE	TAGCGGTCCTTACCTGCGGCG	3' RACE
mAs-F qPCR	TGAAGCCAAGCTGGAGGTAT	qPCR
mAs-R qPCR	GCCAATGTCCCAGTCCTTTA	qPCR
mPhox2b-F qPCR	TCTTCGCTGAGACGCACTAC	qPCR
mPhox2b-R qPCR	GCTCTTGGCCTCTTTGCTCT	qPCR
18S-F qPCR	GGCCGTTCTTAGTTGGTGGA	qPCR
18S-R qPCR	TCAATCTCGGGTGGCTGAAC	qPCR
mPhox2b-F ISH	ATTGAATTCGTGGCTTCCAGTATAACCCG	Probe for ISH
mPhox2b-R ISH	ATTGCGGCCGCCGCCCTTGCCGGGTTCG	Probe for ISH
mPhox2b-As-F ISH	ATTGAATTCCTGCGGCGTACGGACTGCTCTGGT	Probe for ISH

mPhox2b-As-R ISH	ATTGCGGCCGCGAGCTTTACTCGGTTGTTTATTTTAC	Probe for ISH
PAX6-F qPCR	CGCCTATGCCCAGCTTCAC	qPCR
PAX6-R qPCR	GGCAGCATGCAGGAGTATGAG	qPCR
TUBB3-F qPCR	GGCCAAGTTCTGGGAAGTCA	qPCR
TUBB3-R qPCR	CCGAGTCGCCCACGTAGTT	qPCR
BRACHYURY-F qPCR	GGGTCCACAGCGCATGAT	qPCR
BRACHYURY-R qPCR	ATTTTAAGAGCTGTGATCTCCTCGTT	qPCR
NCAM-F qPCR	TCCTGGGAACTGCAGTTTCTCT	qPCR
NCAM-R qPCR	TTTGGCATCTCCTGCCACTT	qPCR
FOXA2-F qPCR	TTCAGGCCCGGCTAACTCT	qPCR
FOXA2-R qPCR	ACCCCCACTTGCTCTCTCACT	qPCR
GATA4-F qPCR	AGCTGGGTAGTTTAGCCAAACG	qPCR
GATA4-R qPCR	TGTGTGACACGGTGAACGAA	qPCR
HPRT1-F qPCR	TTTGCTGACCTGCTGGATTACA	qPCR
HPRT1-R qPCR	GGTCATTACAATAGCTCTTCAGTCTGAT	qPCR
2Bex3-F	TGCTTCACCGTCTCTCCTTCC	PCR (Sanger)
2Bex3-R	TACCCGCTCGCCCACTCG	PCR (Sanger)

Table S2. Chemicals, antibodies, commercial assays, plasmids and software
All chemicals, antibodies, critical commercial assays, plasmids and software used in this paper.

REAGENT	SOURCE	IDENTIFIER
**Antibodies**		
Mouse monoclonal anti-SSEA4	Cell Signaling Technology	Cat#4755; RRID: AB_1264259
Rabbit monoclonal anti-OCT4	Cell Signaling Technology	Cat#2840; RRID: AB_ 2167691
Rabbit monoclonal anti-SOX10	Cell Signaling Technology	Cat#89356; RRID: AB_2792980
Mouse monoclonal anti-PHOX2B (C-3)	Santa Cruz Biotechnology	Cat# sc-376993; RRID: AB_2923265
Mouse monoclonal anti-PHOX2B (B-11)	Santa Cruz Biotechnology	Cat# sc-376997; RRID: AB_ 2813765
Rabbit monoclonal anti-TUBB3	Cell Signaling Technology	Cat#5568; RRID: AB_10694505
Rabbit monoclonal anti-TH	Cell Signaling Technology	Cat#58844; RRID: AB_2744555
Mouse monoclonal anti- βTubulin	Cell Signaling Technology	Cat#86298; RRID: AB_2715541
Donkey anti-rabbit IgG Alexa Fluor 488	Jackson ImmunoResearch	Cat#711-545-152; RRID: AB_2313584
Goat anti-mouse IgG DyLight 549	Jackson ImmunoResearch	Cat#115-505-146; RRID: AB_2341133
Goat anti-mouse IgG (HRP)	Pierce	Cat#1858413
**Chemicals, peptides, and recombinant proteins**		
PowerSYBR® Green PCR Master Mix	Applied Biosystems	Cat#4367659
SYBR™ Select Master Mix	Applied Biosystems	Cat#4472908
FastStart Essential DNA Green Master	Roche	Cat#06402712001
TaqMan ™ Gene Expression Master Mix	Applied Biosystems	Cat#4369016
GoTaq G2 Flexi DNA polymerase	Promega	Cat#M7805
GC-rich PCR system	Roche	Cat#04743784001 Cat#12140306001
Expand High Fidelity PCR system	Roche	Cat#04738250001
ExoSAP-IT™ PCR Product Cleanup Reagent	Applied Biosystems	Cat#78201.1.ML
B-Mercaptoethanol	Sigma-Aldrich	Cat#M-3148
Agarose	Euroclone	Cat#EMR920500
Ethidium Bromide	Sigma-Aldrich	Cat#E-2515
100 bp DNA ladder	New England BioLabs	Cat#N3231S
1000 bp ladder	New England BioLabs	Cat#N3232S
TRIzol™ Reagent	TRIzol™ Reagent	Invitrogen
DAPI	Sigma-Aldrich	Cat#D8417
Paraformaldehyde 8% Aqueous solution, EM Grade	Electron Microscopy Sciences	Cat#157-8-100
Triton™ X-100 Surfact-Amps™ Detergent Solution	Thermo Fisher Scientific	Cat#28314
ProLong™ Glass Antifade Mountant	Invitrogen	Cat#P36982
RPMI 1640, 1x	Corning	Cat#15-041-CV
DMEM High Glucose	Corning	Cat#15-013-CV
DMEM High Glucose	Gibco	Cat#11960044
DMEM-F12	Gibco	Cat#31330-038
Fetal bovine serum	Corning	Cat#35-079-CV
G-418 Sulfate	Gibco	Cat#10131-035
Fugene HD Transfection reagent	Promega	Cat#E2311
Lipofectamine RNAiMAX Transfection Reagent	Invitrogen	Cat#13778075
3-KDG, Etonogestrel	USBiological	Cat#E8797

ATRA	Sigma-Aldrich	Cat#R2625
Gentle Cell Dissociation Reagent	Stem Cell Technologies	Cat#100-0485
Y-27632	Stem Cell Technologies	Cat#72302
Essential 8 FLEX Medium Kit	Gibco	Cat#A2858501
Essential 8 Medium	Gibco	Cat# A1517001
Matrigel Growth Factor Reduced	Corning	Cat#CLS354230
Matrigel hESC-qualified Matrix	Corning	Cat#CLS354277
poly-L-lysine	Sigma-Aldrich	Cat#P2636
EDTA	Invitrogen	Cat#15575020
Essential 6 Medium	Gibco	Cat#A15164-01
SB431542	Cayman Chemical Company	Cat#13031
CHIR99021	Tocris Bioscience	Cat#4423
FGF-2	PeproTech	Cat#100-18B
GlutaMAX 100X	Gibco	Cat#35050-038
Neurobasal Medium	Gibco	Cat#A35829-01
B27 Plus Supplement 50X	Gibco	Cat#A35828-01
N2 Supplement 100X	Gibco	Cat#17502-048
Ascorbic acid	Sigma-Aldrich	Cat#A4034
BMP4	PeproTech	Cat#120-05
BDNF	PeproTech	Cat#450-02
NGF	PeproTech	Cat#450-01
GDNF	PeproTech	Cat#AF-450-10
LiteAblot EXTEND	Euroclone	Cat#EMP013001
Hs-PHOX2B-C2	ACD	Cat#567701-C2
Hs-PHOX2B-AS1-01	ACD	Cat#1245601-C1
**Critical commercial assays**		
RNeasy Micro Kit	Qiagen	Cat#74004
RNeasy Mini Kit	Qiagen	Cat#74104
QIAshredder ™	Qiagen	Cat#79654
NucleoSpin gel and Clean-up PCR kit	Macherey & Nagel	Cat#740609.250
pCRII-TOPO TA	Invitrogen	Cat#45-0640
FirstChoice® RLM RACE Kit	Invitrogen	Cat#AM1700
CytoTune-iPS 2.0 Sendai kit	Invitrogen	Cat#A16517
STEMdiff™ Trilineage Differentiation Kit	Stem Cell Technologies	Cat#05230
Dual-Luciferase® Reporter Assay System	Promega	Cat#E1910
RNAscope Multiplex Fluorescent Reagent Kit v2	ACD	Cat#323100
High-Capacity RNA-to-cDNA™ Kit	Applied Biosystems	Cat#4374967
BigDye™ Terminator v1.1 Cycle Sequencing Kit	Applied Biosystems	Cat# 4337450
GoScript Reverse Transcription System	Promega	Cat#A5001
SuperScript ™ III First-Strand Synthesis System	Invitrogen	Cat#18080051
**Oligonucleotides**		
Table S1 (primers and gapmeRs)		
Table S4 (TaqMan assays)		
**Recombinant DNA**		
pCRII	Invitrogen	Cat#45-0640
pGL4.74 [hRluc/TK] vector	Promega	Cat#E6921
phRG-B vector	Promega	Cat#E6281
pGL4.10[luc2]	Promega	Cat#E6651
PHOX2B-AS1 promoter +2933/+366	This paper	
PHOX2B-AS1 promoter +1475/+366	This paper	
PHOX2B-AS1 promoter +806/+366	This paper	

pcDNA3.1 Myc His C	Invitrogen	Cat#V80020
PHOX2B WT in pCDNA3.1	Bachetti et al. 2005 [36]	
PHOX2B +7 in pCDNA3.1	Di Lascio et al. 2013 [37]	
PHOX2B +13 in pCDNA3.1	Di Lascio et al. 2013 [37]	
**Software and algorithms**		
SRA Toolkit v3.1.0	NCBI	https://github.com/ncbi/sra-tools
Salmon v1.10.3	Patro et al. 2017	https://github.com/COMBINE-lab/salmon
UCSC Genome Browser	NIH	http://genome.ucsc.edu
Ensembl	EMBL-EBI	https://www.ensembl.org
NCBI	NIH	https://www.ncbi.nlm.nih.gov
Clustal Omega	EMBL-EBI	http://www.clustal.org/ https://www.ebi.ac.uk/jdispatcher/msa/clustalo
ExPASy	Duvaud et al. 2021	http://web.expasy.org/translate/
ATGpr	Helix Research Institute	http://atgpr.dbcls.jp/
Photoshop CS6	Adobe	https://www.adobe.com
Affinity Photo 2.6.2	Serif	https://affinity.serif.com/en-gb/photo/
Image Lab v6.0	Bio-rad	https://www.bio-rad.com/
Zeiss ZEN 3.9	Carl Zeiss Microscopy GmbH	https://www.zeiss.com/microscopy/en/home.html
LASX Office 1.4.5 27713	Leica Microsystems	https://www.leica-microsystems.com/
Biorender	Biorender	www.biorender.com
Prism, v10.3.0	GraphPad Prism	https://www.graphpad.com/features
Design and Analysis v2 (DA2)	Applied Biosystem	https://apps.thermofisher.com
IGV	Robinson et al. 2011	http://www.broadinstitute.org/igv/
Sequencher 5.0 software	Gene Codes Corporation	https://www.genecodes.com/
Finch TV 1.4.0 software	Geospiza Inc.	https://finchtv.software.informer.com/1.4/#google_vignette

Table S3. Differentiation media

A table listing the components of the differentiation media along with their final concentrations. CHIR99021: GSK3β inhibitor and WNT pathway activator; SB431542: inhibitor of TGFβ receptors ALK4/5/7; FGF-2: basic fibroblast growth factor; BMP4: bone morphogenic protein 4; B27: optimized serum-free supplement used for the maintenance and maturation of stem cell-derived neurons; N2 chemically-defined, serum-free supplement based on Bottenstein's N-1 formulation; NGF: nerve growth factor; BDNF: brain-derived neurotrophic factor; GDNF: glial cell-derived neurotrophic factor

**Induction medium 1 (Days 0-3)**		
**Reagent**	**Final conc.**	**Source / Identifier**
Essential 6 Medium	-	Gibco Cat#A15164-01
SB431542	10 µM	Cayman Chemical Company Cat#13031
CHIR99021	3 µM	Tocris Bioscience Cat#4423
FGF-2	8 ng/mL	PeproTech Cat#100-18B
GlutaMAX 100X	1X	Gibco Cat#35050-038
**Trunk induction medium (Days 3-10)**		
Essential 6 medium	-	Gibco Cat#A15164-01
SB431542	10 µM	Cayman Chemical Company Cat#13031
CHIR99021	3 µM	Tocris Bioscience Cat#4423
FGF-2	8 ng/mL	PeproTech Cat#100-18B
Retinoic acid	1 µM	Sigma-Aldrich Cat#R2625
GlutaMAX 100X	1X	Gibco Cat#35050-038
**SAP induction medium (Days 10-14)**		
Neurobasal Medium	-	Gibco Cat#A35829-01
B27 Plus Supplement 50X	1X	Gibco Cat#A35828-01
N2 Supplement 100X	1X	Gibco Cat#17502-048
GlutaMAX 100X	1X	Gibco Cat#35050-038
Ascorbic acid	200 µM	Sigma-Aldrich Cat#A4034
BMP4	50 ng/mL	PeproTech Cat#120-05
FGF-2	8 ng/mL	PeproTech Cat#100-18B
**symNs maturation medium (Day 14-onwards)**		
Neurobasal Medium	-	Gibco Cat#A35829-01
B27 Plus Supplement 50X	1X	Gibco Cat#A35828-01
N2 Supplement 100X	1X	Gibco Cat#17502-048
GlutaMAX 100X	1X	Gibco Cat#35050-038
Ascorbic acid	200 µM	Sigma-Aldrich Cat#A4034
BDNF	10 ng/mL	PeproTech Cat#450-02
NGF	10 ng/mL	PeproTech Cat#450-01
GDNF	10 ng/mL	PeproTech Cat#AF-450-10

Table S4. List of TaqMan assays used in this study
This table includes all TaqMan gene expression assays employed (Applied Biosystems, Cat#4331182), along with their corresponding assay IDs and target genes.

TaqMan assays	
**Target**	**Assay ID**
PHOX2B	Hs00243679_m1
DBH	Hs01089840_m1
CHAT	Hs00252848_m1
HOXC8	Hs00224073_m1
HOXC9	Hs00396786_m1
SOX10	Hs00366918_m1
ISL1	Hs00158126_m1
ASCL1	Hs00269932_m1
PRPH	Hs00196608_m1
GAPDH	Hs99999905_m1
